# Supplementary material for: Posterior lamellar versus bilamellar tarsal rotation surgery for trachomatous trichiasis: Long-term outcomes from a randomised controlled trial
Source: eClinicalMedicine. 2019 Nov 1;17:100202. doi: 10.1016/j.eclinm.2019.10.015 (PMC6933225; doi:10.1016/j.eclinm.2019.10.015)
Supplement: Supplementary Data S1 — Supplementary Raw Research Data. This is open data under the CC BY license http://creativecommons.org/licenses/by/4.0/ [file mmc1.zip › S2589537019302019.zip!/Responses to Editorial Requirments.docx]

Dear Dr Colosio,

Ms. Ref. No.:  ECLINM-D-19-00219

Title: Posterior lamellar versus bilamellar tarsal rotation surgery for trachomatous trichiasis: long-term outcomes from a randomised controlled trial

Thank you very much for inviting us to revise our manuscript. We are grateful for the editorial office for sending our manuscript to a second statistical reviewer.

We have revised our manuscript based on the RTC Lancet guidelines and uploaded a clean copy and a copy with alterations in track changes.

Yours sincerely,

Esmael Habtamu
