# Supplementary material for: Posterior lamellar versus bilamellar tarsal rotation surgery for trachomatous trichiasis: Long-term outcomes from a randomised controlled trial
Source: eClinicalMedicine. 2019 Nov 1;17:100202. doi: 10.1016/j.eclinm.2019.10.015 (PMC6933225; doi:10.1016/j.eclinm.2019.10.015)
Supplement: Supplementary Data S1 — Supplementary Raw Research Data. This is open data under the CC BY license http://creativecommons.org/licenses/by/4.0/ [file mmc1.zip › S2589537019302019.zip!/BLTR v PLTR- 4yr FU MoST Approval Letter.pdf]

ቁጥር
Ref.No. 3.20/273/2017
ቀን
Date. Nov. 1, 2017

To: The Carter Center, Ethiopia

Addis Ababa

Subject: Acceptance Letter of Amendment Request

Dear Sir/Madam /Mr./Mrs./Dr.

We are writing this letter in reference to your amendment request letter Ref. no 1201/2017 dated 21, September 2017.

After having in depth review of your request on **"A Randomized Controlled Trial of Bilamellar Tarsal Rotation (BLTR) Vs. Posterior Lamellar Tarsal Rotation (PLTR) Surgery for Tracomatous Trichiasis and Trichiasis Surgery Impact Study"** the National Research Ethics Review Committee has accepted your amendment request for one year from **(November 1, 2017- October 31, 2018).** This is, therefore, to notify that the ethical approval is renewed and your group can proceed in accordance to the latest approved document. Please ensure that you submit a biannual report and an annual renewal application 30 days prior to expire date. We are confident that you as PI of the project and your esteemed organization will monitor the ethical implication of the project as it is stipulated in the latest approved document.

With Regards,

Awol Hussein Mohammed
Research Ethics Directorate
Acting Director

The Federal Democratic Republic of Ethiopia Ministry of Science and Technology

CC

- **H-E the Minister**
- **Chairperson, NRERC**
- **Dr. Esmael Habtamu Ali (PI)**
