# Supplementary material for: Posterior lamellar versus bilamellar tarsal rotation surgery for trachomatous trichiasis: Long-term outcomes from a randomised controlled trial
Source: eClinicalMedicine. 2019 Nov 1;17:100202. doi: 10.1016/j.eclinm.2019.10.015 (PMC6933225; doi:10.1016/j.eclinm.2019.10.015)
Supplement: Supplementary Data S1 — Supplementary Raw Research Data. This is open data under the CC BY license http://creativecommons.org/licenses/by/4.0/ [file mmc1.zip › S2589537019302019.zip!/Emory - Amendment 5_ BLTRvPLTR_Approved._2017pdf.pdf]

EMORY UNIVERSITY | Institutional Review Board

TO: Elizabeth Callahan, MD
Principal Investigator
*The Carter Center DeptApproval

DATE: September 18, 2017

RE: **Notification of Amendment Approval**
AM5_IRB00067868
IRB00067868
Randomised controlled trial of Bilamellar Tarsal Rotation (BLTR) verses Posterior Lamellar Tarsal Rotation (PLTR) surgery for trachomatous trichiasis

Thank you for submitting an amendment request. The Emory IRB reviewed and approved this amendment under the expedited review process on **09/15/2017**. This amendment includes the following:

- Changes to Consent Form:
  - Information & Consent - BLTR v PLTR- Cases-4y Fu_v1_09Aug17
  - Information & Consent - Impact Study - Controls-4y FU_v1 09Aug17
- Changes to Protocol Document:
  - 1. RCT BLTR v PLTR - Detailed Study Protocol - Amended 12AUG2017

Important note: If this study is NIH-supported, you may need to obtain NIH prior approval for the change(s) contained in this amendment before implementation. Please review the NIH policy directives found at the following links and contact your NIH Program Officer, NIH Grants Management Officer, or the Emory Office of Sponsored Programs if you have questions.

Policy on changes in active awards: http://grants.nih.gov/grants/guide/notice-files/NOT-OD-12-129.html

Policy on delayed onset awards: http://grants.nih.gov/grants/guide/notice-files/NOT-OD-12-130.html

In future correspondence with the IRB about this study, please include the IRB file ID, the name of the Principal Investigator and the study title. Thank you.

Sincerely,

Leslie J. Garrett
Sr. Research Protocol Analyst
This letter has been digitally signed

CC	Bloch	Nina	*The Carter Center DeptApproval
	Stewart	Aisha	*The Carter Center DeptApproval

Emory University IRB
1599 Clifton Road, 5th Floor - Atlanta, Georgia 30322
Tel: 404.712.0720 - Fax: 404.727.1358 - Email: irb@emory.edu - Web: http://www.irb.emory.edu/
An equal opportunity, affirmative action university
