# Supplementary material for: Posterior lamellar versus bilamellar tarsal rotation surgery for trachomatous trichiasis: Long-term outcomes from a randomised controlled trial
Source: eClinicalMedicine. 2019 Nov 1;17:100202. doi: 10.1016/j.eclinm.2019.10.015 (PMC6933225; doi:10.1016/j.eclinm.2019.10.015)
Supplement: Supplementary Data S1 — Supplementary Raw Research Data. This is open data under the CC BY license http://creativecommons.org/licenses/by/4.0/ [file mmc1.zip › S2589537019302019.zip!/FMHCA Approval.pdf]

የኢትዮጵያ የምግብ፣የመድኃኒትና የጤና ክብካቤ አስተዳደርና ቁጥጥር ባለስልጣን
FOOD, MEDICINE AND HEALTH CARE ADMINISTRATION AND CONTROL AUTHORITY OF ETHIOPIA

ቁጥር Ref.No. 02/6/33/27
ቀን Date 11 JAN 2018

To: Carter Center Ethiopia
Addis Ababa

Subject: Clinical Trial Authorization

It is hereby certified that the food, Medicine and Health Care Administration and Control Authority, being the authority responsible by the law to authorized and monitor clinical trial conducted in the country (proclamation 661/2009), has officially authorized the conduct of clinical trial entitled **"Randomised Controlled trial of Bilamellar Tarsal Rotation (BLTR) versus Lamellar Tarsal Rotation (PLTR) surgery for trachomatous trichiasis surgery impact study four years follow up"**. The clinical trial authorization is subject to the following conditions:

- Initiation, progress and termination/end of trial reports shall be submitted to the authority.
- Any adverse events especially serious adverse event/s or deaths and progress report should be reported to the authority as per the specified time on the guideline
- The authority shall be informed of any decision to discontinue the clinical trial (if it is found necessary and the reason of such action will be disclosed to the applicant)
- The clinical trial should be conducted according to the protocol and if any amendment required, the amendment should be approved by the authority before implementation.
- The authority shall inspect the trial site at any time for compliance of the trial for Good Clinical Trial Practice (GCP) and the protocol.

With best regards,

Habtamu Beyene
DIRECTOR, MEDICINE REGISTRATION AND LICENSING DIRECTORATE

CC:

- Dr. Esmael Habtamu (PI)
  Bahir Dar
- Medicine registration and Licensing Directorate
  EFMHACA

ፋክስ/Fax: 251 552 13 92 P.O.Box: 5681 Tel. 251 552 41 22/52 41 23
E-mail: regulatory@fmhaca.gov.et www.fmhaca.gov.et
መልስ በሚሰጡበት ጊዜ የእኛን ደብዳቤ ቁጥር ይጥቀሱ
IN REPLY REFER TO OUR Ref.No.
