# Supplementary material for: Posterior lamellar versus bilamellar tarsal rotation surgery for trachomatous trichiasis: Long-term outcomes from a randomised controlled trial
Source: eClinicalMedicine. 2019 Nov 1;17:100202. doi: 10.1016/j.eclinm.2019.10.015 (PMC6933225; doi:10.1016/j.eclinm.2019.10.015)
Supplement: Supplementary Data S1 — Supplementary Raw Research Data. This is open data under the CC BY license http://creativecommons.org/licenses/by/4.0/ [file mmc1.zip › S2589537019302019.zip!/LSHTM - Amedment Approval BLTR v PLTr_2017.pdf]

London School of Hygiene & Tropical Medicine
Keppel Street, London WC1E 7HT
United Kingdom
Switchboard: +44 (0)20 7636 8636

www.lshtm.ac.uk

LONDON SCHOOL of HYGIENE &TROPICAL MEDICINE

Observational / Interventions Research Ethics Committee

Prof Matthew Burton
Professor of International Eye Health
Department of Clinical Research (CRD)
LSHTM

19 September 2017

Dear Prof Matthew

Study Title: Randomised controlled trial of Bilamellar Tarsal Rotation (BLTR) verses Posterior Lamellar Tarsal Rotation (PLTR) surgery for trachomatous trichiasis and trichiasis surgery impact study

LSHTM Ethics Ref: 6424 - 2

Thank you for your application for the above amendment to the existing ethically approved study and submitting revised documentation. The amendment application has been considered by the Interventions Committee.

Confirmation of ethical opinion

On behalf of the Committee, I am pleased to confirm a favourable ethical opinion for the above amendment to research on the basis described in the application form, protocol and supporting documentation as revised, subject to the conditions specified below.

Conditions of the favourable opinion

Approval is dependent on local ethical approval for the amendment having been received, where relevant.

Approved documents

The final list of documents reviewed and approved by the Committee is as follows:

Document Type	File Name	Date	Version
Other	Information & Consent - BLTR v PLTR- Cases-4y Fu_v1_09Aug17	09/08/2017	1
Other	Information & Consent - Impact Study - Controls-4y FU_v1 09Aug17	09/08/2017	1
Other	1. RCT BLTR v PLTR - Detailed Study Protocol - Amended 12AUG2017	12/08/2017	2

After ethical review

The Chief Investigator (CI) or delegate is responsible for informing the ethics committee of any subsequent changes to the application. These must be submitted to the Committee for review using an Amendment form. Amendments must not be initiated before receipt of written favourable opinion from the committee.

The CI or delegate is also required to notify the ethics committee of any protocol violations and/or Suspected Unexpected Serious Adverse Reactions (SUSARs) which occur during the project by submitting a Serious Adverse Event form.

An annual report should be submitted to the committee using an Annual Report form on the anniversary of the approval of the study during the lifetime of the study.

At the end of the study, the CI or delegate must notify the committee using an End of Study form.

All aforementioned forms are available on the ethics online applications website and can only be submitted to the committee via the website at: http://leo.lshtm.ac.uk

Additional information is available at: www.lshtm.ac.uk/ethics

Yours sincerely,

Professor John DH Porter
Chair

ethics@lshtm.ac.uk
http://www.lshtm.ac.uk/ethics/

Improving health worldwide
