# Supplementary material for: Posterior lamellar versus bilamellar tarsal rotation surgery for trachomatous trichiasis: Long-term outcomes from a randomised controlled trial
Source: eClinicalMedicine. 2019 Nov 1;17:100202. doi: 10.1016/j.eclinm.2019.10.015 (PMC6933225; doi:10.1016/j.eclinm.2019.10.015)
Supplement: Supplementary Data S1 — Supplementary Raw Research Data. This is open data under the CC BY license http://creativecommons.org/licenses/by/4.0/ [file mmc1.zip › S2589537019302019.zip!/1. RCT BLTR v PLTR - Detailed Study Protocol - Amended 12AUG2017.docx]

**Randomised controlled trial of Bilamellar Tarsal Rotation (BLTR) verses Posterior Lamellar Tarsal Rotation (PLTR) surgery for trachomatous trichiasis and trichiasis surgery impact study**

**Study Proposal**

**LSHTM Principal Investigator:** Matthew Burton^1^

**Ethiopian Principal Investigator / PhD Student:** Esmael Habtamu Ali^1,2^

**Co-Investigators:**

Zerihun Tadese^2^

Bizuayehu Gashaw^3^

Kelly Callahan^4^

Saul Rajak^1^

David Macleod^1^

Helen Weiss^1^

^1^ London School of Hygiene & Tropical Medicine, UK

^2^ The Carter Centre, Ethiopia

^3^ Amhara Regional Health Bureau, Ethiopia

^4^ Trachoma Control Programme, Carter Centre, Atlanta

Table of Contents

[Summary	5](#_Toc490147489)

[Four-year Follow-up Summary	6](#_Toc490147490)

[Background	6](#_Toc490147491)

[Study Plan	6](#_Toc490147492)

[Anticipated Impact	6](#_Toc490147493)

[Introduction	7](#_Toc490147494)

[Trachoma	7](#_Toc490147495)

[Trachoma in Ethiopia	7](#_Toc490147496)

[Prevention of Blindness from Trachoma	7](#_Toc490147497)

[Trichiasis Surgery	8](#_Toc490147498)

[Reasons for Recurrent Trichiasis	8](#_Toc490147499)

[The impact of Trichiasis and Trichiasis Surgery	10](#_Toc490147500)

[Rationale	12](#_Toc490147501)

[Study Objectives	13](#_Toc490147502)

[Four-year follow-up study objectives	13](#_Toc490147503)

[Methods	14](#_Toc490147504)

[Study Design	14](#_Toc490147505)

[Study Area and Population	14](#_Toc490147506)

[Research Partnership	15](#_Toc490147507)

[Sample Size	15](#_Toc490147508)

[Trichiasis cases	15](#_Toc490147509)

[Unexposed/Controls	16](#_Toc490147510)

[Informed Consent	17](#_Toc490147511)

[Baseline Assessment	18](#_Toc490147512)

[Randomisation, Allocation Concealment and Masking	19](#_Toc490147513)

[Trichiasis Surgeon Standardization	20](#_Toc490147514)

[Surgical Treatment	21](#_Toc490147515)

[Follow-up Assessments	22](#_Toc490147516)

[Fourth Year Follow-up Assessments	23](#_Toc490147517)

[Informed Consent	23](#_Toc490147518)

[Questionnaire	24](#_Toc490147519)

[Clinical examination	24](#_Toc490147520)

[Data Analysis	24](#_Toc490147521)

[Clinical outcomes at four years	26](#_Toc490147522)

[Fourth year follow-up clinical data analysis	26](#_Toc490147523)

[Four-year follow-up mental health data analysis	26](#_Toc490147524)

[Study Duration	27](#_Toc490147525)

[Ethics Approval	27](#_Toc490147526)

[Study Benefits	27](#_Toc490147527)

[Risk Assessment	29](#_Toc490147528)

[Study Monitoring	29](#_Toc490147529)

[Study Management	30](#_Toc490147530)

[References	32](#_Toc490147531)

[Appendices	34](#_Toc490147532)

[Appendix 1: Standard Operating Procedure for Taking Informed Consent	34](#_Toc490147533)

[Appendix 2: Standard Operating Procedure for Clinical Examination	36](#_Toc490147534)

[Appendix 3: BLTR Surgical Procedure	41](#_Toc490147535)

[Appendix 4: PLTR Surgical Procedure	44](#_Toc490147536)

[Appedix 5: Adverse Event Reporting Protocol	46](#_Toc490147537)

[Appendix 6: SAE reporting Format	47](#_Toc490147538)

**Glossary of Abbreviations**

| BLTR | Bilamellar Tarsal Rotation Surgery |
| --- | --- |
| DSMC | Data Safety Monitoring Committee |
| HRQoL | Health Related Quality of Life |
| LMICs | Low and Middle Income Countries |
| LSHTM | London School of Hygiene & Tropical Medicine |
| LSMS | Living Standard Measurement Survey |
| PBL | Prevention of Blindness |
| PIQ | Pain Impact Questionnaire |
| PLTR | Posterior Lamellar Tarsal Rotation Surgery |
| QoL | Quality of Life |
| RCT | Randomised Controlled Trial |
| TSC | Trial Steering Committee |
| TT | Trachomatous Trichiasis |
| VRQoL | Vision Related Quality of Life |
| WHO | World Health Organization |
| WHOQOL-BREF | World Health Organization Quality of Life - Brief version of QoL measure |

# Summary

| **BACKGROUND** | Despite being a WHO priority research question, the two commonly used trichiasis surgery procedures (BLTR and PLTR) have not been adequately compared and it is not known which of these procedures provide the best results under operational conditions. There is currently very limited data on the impact of trichiasis and trichiasis surgery on the quality of life and economic welfare of patients. |
| --- | --- |
| **AIMS** | To examine whether BLTR or PLTR gives better results for the treatment of trachomatous trichiasis under operational conditions and to measure the impact of trichiasis surgery on the life and economic welfare of patients |
| **DESIGN** | Prospective, double-masked randomised controlled trial of two alternative surgical treatments (BLTR and PLTR) for individuals with previously un-operated trachomatous trichiasis; with an additional cohort study nested within the RCT for the trichiasis surgery impact study |
| **OUTCOME MEASURES** | *Primary outcome measure:* recurrent trichiasis, defined as one or more lashes touching the eye or clinical evidence of epilation at either the six or twelve month assessments or a history of repeat surgery during the last one year after the baseline surgery  *Secondary outcome measures will include:*   - Severity of recurrent trichiasis (number of lashes) and entropion - Change in corneal opacification (by comparison of photographs) - Change in visual acuity (LogMAR) - Conjunctival inflammation - Complication rates - Impact of trichiasis and trichiasis surgery on pain, vision and health related quality of life, activity and household poverty |
| **POPULATION** | The study will be conducted in Amhara Region, Ethiopia. About 1000 trachomatous trichiasis patients (one or more lashes touching the eye ball or with evidence of epilation) and 200 controls without trichiasis will be recruited through community based trichiasis surgery outreaches and village level screening. |
| **ELIGIBILITY** | ***Inclusion criteria:***   1. Trachomatous Trichiasis: trichiasis with tarsal conjunctival scarring 2. Adults (≥18 years) 3. Agree to be randomly allocated to one of the two alternative operative procedures 4. Agree to re-examination at six and twelve months after surgery   ***Exclusion criteria:***   1. Previous eyelid surgery. 2. Other eyelid pathology.   **Unexposed cases for the impact study:** People without trichiasis and location, sex and age (within five years) matched with 200 consecutive trichiasis cases (exposed). |
| **TREATMENT** | Cases (trichiasis patients) will be randomly allocated and receive surgical treatment with one of the surgical procedures (BLTR or PLTR). Currently both of these operations are widely used in Ethiopia and are approved for the treatment of TT by the WHO. Participants will be followed for 1 year at three time points. |
| **duration** | The anticipated overall project duration is about 4 years. |

# Four-year Follow-up Summary

## Background

Trachomatous Trichiasis (TT), the blinding stage of trachoma, is mainly treated surgically. The two most commonly used surgical procedures to treat TT are the Posterior Lamellar Tarsal Rotation (PLTR) and the Bilamellar Tarsal Rotation Surgery. We recently conducted a randomised, controlled, single masked clinical trial for their relative effectiveness, published in Lancet Global Health in 2016.^1^

One thousand individuals who had upper lid trachomatous trichiasis with one or more eyelashes touching the eye or evidence of epilation, in association with tarsal conjunctival scarring were enrolled into the trial from West Gojam Zone Amhara Region, Ethiopia between February and May 2014. These were randomly allocated and received surgical management either using BLTR or PLTR surgery, and then were followed at 6 and 12 months by assessors masked to allocation. The result of the trial from the one year data showed that cumulative recurrent trichiasis by 12 months was more frequent in the BLTR group than in the PLTR group (110/496 [22%] *vs* 63/496 [13%]). We achieved 98% follow-up at one year. Because of this, WHO and international guidance on the surgical treatment of choice has been updated, shifting away from the BLTR being the treatment of choice to a neutral position overall and a preference for new trainees to be taught the PLTR.

However, there is currently no data on the long-term outcome of these two surgical procedures. Some studies have reported that rate of postoperative TT might increase from about 20% at one year to as much as 60% at 3 years after surgery.^2^ There is currently much international interest to see the long-term outcome of the two surgical procedures compared in this project. Therefore, we would like to follow-up and examine the trial participants of the above trial 4 years after enrolment to answer the following questions: (1) Are the results of PLTR and BLTR surgical procedures different 4 years after surgery? (2) Does eyelid contour abnormality (ECA) resolve or regress through time? (3) What are the factors influencing long-term outcomes in PLTR and BLTR surgery? (4) What is the impact of Trichiasis surgery and poor surgical outcome on mental health?

## Study Plan

Trial participants will be informed about the need to do long-term follow-up and would be asked for their consent to participate. An independent accessor masked to allocation will examine the operated eyes of the participants using 2·5× binocular loupes and torch, and the detailed WHO FPC Trachoma Grading System. The primary outcome would be rate of postoperative trachomatous trichiasis (PTT) at four years, while secondary outcomes would include rate and regression of ECA, risk factors for PTT, change in corneal opacity and vision, retrospective analyses on the impact of TT, TT surgery, and PTT on mental health. The mental health status of the trichiasis cases will be assessed using two mental health assessment tools (Patient Health Questionnaire - 9 (PHQ – 9), and Self Reporting Questionnaire (SRQ20)), in-depth interviews and focus group discussions.

## Anticipated Impact

There are still 3.2 million un-operated cases of TT globally. These would need to be operated with the safest and most successful surgical procedure. The results of the one year follow-up for the above trial has already influenced global policy leading the WHO to recommend the use of the PLTR for training of new TT surgeons.^3^ However, it is possible that outcomes might change few years after surgery. The data from this proposed long-term follow-up will provide additional concrete evidence on what would happen to those operated using either of the surgical procedures some years after surgery.

# Introduction

## Trachoma

Trachoma is the leading infectious cause of blindness worldwide.^4^ It is a chronic kerato-conjunctivitis caused by *Chlamydia trachomatis* (*Ct*). Trachoma begins in childhood with recurrent episodes of follicular/papillary conjunctivitis (active trachoma). Chronic inflammation results in immuno-pathologically mediated conjunctival scarring (TS), which causes eyelid in-turning (entropion) with eyelashes scratching the eye, which is known as trichiasis (TT). Eventually sight is lost as irreversible corneal opacification develops (CO).

There are around 1.3 million people blind and 6 million severely visually impaired from trachoma.^4^ About 8 million have un-treated trichiasis and are at significant risk of losing sight.^5^ The impact of blinding trachoma on the individual is devastating. The blindness (unlike that of cataract) is irreversible and can be very painful. Trachoma has major social and economic consequences for effected families and communities.

## Trachoma in Ethiopia

Ethiopia is considered to have one of the most serious trachoma problems in the world. It is estimated that 30% of the African continent’s burden of trachoma is in Ethiopia. Trachoma is the second largest cause of blindness after cataract accounting 11.5% of national blindness.^6^ It is estimated that among the population of 77 million Ethiopians (2007), 65 million live in trachoma endemic areas and more than 138,000 people are blind from the disease. The prevalence of clinically active trachoma and detection of *C.trachomatis* infection in children is approximately 40-50%.^3;4^ Potentially blinding trichiasis affects 3-5% of the adult population, estimated at 1.9 million people.^3;5^

Amhara Region has a disproportionately large burden of Ethiopia’s trichiasis, bearing an estimated 45% of the total. A population based prevalence survey conducted in 16 randomly selected clusters in each of the ten zones, showed the prevalence of active trachoma ranges from 12.6% to 60.1% (in children under 10 years) and the prevalence of TT from 2.4% to 10% (in adults).^7^ In 2011, it was estimated that there were 480,000 individuals with un-operated trichiasis in the region (Amhara regional Health Bureau – Carter Center Report).

## Prevention of Blindness from Trachoma

The WHO Alliance for the Global Elimination of Blinding Trachoma by 2020 (GET2020) recommends the use of the ***SAFE* *Strategy*** for trachoma control.^8^ This involves **S**urgery for trichiasis, together with **A**ntibiotics (azithromycin or tetracycline), **F**acial cleanliness and **E**nvironmental improvements to suppress chlamydial infection and transmission. However, implementing *SAFE* presents considerable challenges.

Surgery reduces the risk of blindness, as TT is the main risk factor for corneal opacification.^9-11^ However, TT frequently recurs after surgery for reasons that are poorly understood.^2^ The *AFE* components focus on controlling ocular *Ct* infection within endemic communities, to prevent future scarring complications in younger generations. However, established conjunctival scarring in adults appears to be progressive even in regions where the prevalence of active trachoma and *Ct* has been low for some time.^12-14^

## Trichiasis Surgery

Surgical correction of trichiasis is performed because it is believed to reduce the risk of blindness by limiting the abrasive damage of the eyelashes rubbing on the cornea. The surgery usually involves cutting through the scarred eyelid, followed by rotation and suturing of the eyelid in the corrected, everted (turned out) position.^10^ Patients also benefit from a marked reduction in the pain they experience and may have some improvement in vision.^11;12^ TT surgery is usually performed in health centres close to where patients live by nurses who have only limited surgical training. Unfortunately, trichiasis frequently returns during the months and years following the surgery. Studies of the outcome of trichiasis surgery have consistently found trichiasis recurrence rates between 10% at 3 months and 60% at 3 years after surgery, with an average of around 20% at one year.^11-21^

## Reasons for Recurrent Trichiasis

A number of factors may contribute to the development of recurrent trichiasis following corrective surgery: type of surgical procedure used, surgeon’s ability, severity of preoperative eyelid scarring, infection and ongoing conjunctival inflammation. Recurrent trichiasis after surgery is a major problem in the prevention of blinding trachoma. It is therefore important to understand the reasons for surgical failure and to develop practical treatment strategies to reduce the long-term risk of blindness. Some of these factors are discussed below.

#### Surgical Method

Many different surgical procedures have been used to correct TT, suggesting that no “ideal” solution exists.^2^^,^^15^^,^^16^ The commonest operations in use, which are all approved by the WHO, are the Bilamellar Tarsal Rotation (BLTR), the Trabut / Posterior Tarsal Rotation (PLTR) and the Cuenod Nataf procedure.^15;23^ Several surgical procedures have been compared to the BLTR in randomised controlled trials (RCT).^17^^,^^18^ These found that, of the operation types compared, the BLTR procedure had the lowest TT recurrence rate (20% at one year), leading the WHO to recommend it. Although, it is important to note that the widely used PLTR/Trabut was not one of the operation types included in these earlier trials. Subsequently, the lowest reported recurrence rates were from the STAR trial in Ethiopia using BLTR (+/- oral azithromycin), ranging between 5.8% and 10.3% at one-year, although other trials have reported higher rates following BLTR surgery (~20%).^2^^,^^18^^,^^19^ Our recent RCTs in Ethiopia used PLTR, with an overall recurrence rate of 18.4% at one-year.^20^^,^^21^ Importantly, surgeons performing surgery in these three RCTs were selected and standardised by the same Ethiopian ophthalmologist.^20^^,^^22^

Both the BLTR and PLTR procedures are used extensively in Ethiopia.^23^ Previously a randomised trial comparing the BLTR and Trabut/PLTR conducted by ophthalmologists in a teaching hospital in Ethiopia found equivalent outcomes at three months, however, this earlier study was not powered to detect a difference.^24^ It is possible that these alternative techniques, which give comparable results when used by a few very experienced surgeons in a formal study, may give quite different results in the “real world” situation, when performed by less experienced personnel.

The relative effectiveness under operational conditions has been identified as a research priority question by the WHO GET2020 Alliance.^25^

#### Inter-surgeon Variation

A number of studies have found that the results of surgery can vary between different surgeons. Under operational conditions this has ranged up to >50% recurrence at one year for some surgeons.^26^ Even under tightly standardised clinical trial settings, when surgeons are adhering to the procedures, there are some variations in the outcome, suggesting that there are subtle differences between individual surgeons in how they are performing the operations.^19^^,^^27^^,^^28^

#### Infection

Recurrent infection with *Chlamydia trachomatis* may contribute to progressive conjunctival scarring and recurrent trichiasis following surgery, although evidence for this is very limited.^11;13;19;25^ It was suggested that enhanced antibiotic control of infection at the time of and following surgery might improve the outcome. In a study using the antibiotic Azithromycin at the time of surgery conducted in The Gambia (a country with low levels of endemic trachoma), this intervention did not reduce the recurrence rate.^11^ However, in Ethiopia, a country with high levels of Chlamydial infection, Azithromycin appeared to reduce the risk of recurrence of trichiasis after surgery at one year but not at three years.^21^

A strong association has been found between trichiasis and conjunctival bacterial infection (other than *Chlamydia trachomatis*) before and after surgery.^11;13;26^ A wide range of pathogenic organisms have been isolated from the conjunctiva of trichiasis patients, the commonest being *Streptococcus pneumoniae*. The isolation rate increases with increasing severity of trichiasis (number of lashes touching the globe). A year after surgery for trichiasis the bacterial isolation rate was found to be half, suggesting that reducing the burden of trichiasis reduces risk of bacterial infection.^11^

#### Inflammation and Scarring

Persistent severe conjunctival inflammation in children with trachoma is associated with the subsequent development of the scarring complication of trachoma in later life (conjunctival scarring, trichiasis and corneal opacification).^27;28^ Clinically apparent conjunctival inflammation is frequently found in people with trichiasis.^11;13;19;26;29^ It is not known what drives this persistent inflammation in individuals with scarring and trichiasis. It is likely that chronic conjunctival inflammation probably produces progressive scarring of the eyelids leading to trichiasis and corneal opacification both before and after surgery.

Increased expression of pro-inflammatory cytokines such as Interleukin-1β and Tumour Necrosis Factor-α has been found in children with active trachoma.^32;33^ These probably activate matrix metalloproteinases (MMP), which are enzymes that degrade the extracellular matrix, promote inflammatory cell infiltration and facilitate tissue remodelling and scar formation.

In conjunctival surgery, poor outcomes develop because of excessive inflammation/fibrosis during initial wound healing. There are four overlapping phases: Haemostasis, Inflammation, Proliferation, and Remodelling. An imbalance can lead to scarring, primarily mediated by fibroblasts and Matrix Metalloproteinase (MMP) activity. Similar wound healing processes probably occur following trichiasis surgery and may account for a significant component of recurrence within the first few months following surgery.

Evidence of altered tissue remodelling factors are found in recurrent TT, which could favour scarring.^29^ In a recent study, recurrent trichiasis was consistently associated with increased *S100A7* expression.^30^ Much more work is still needed to understand these processes, which would require intensive follow-up and sampling during early weeks following surgery. If MMPs or other mediators are found to be important in the development of blinding trachoma and particularly in the recurrence of trichiasis after surgery, then treatments that block the activity of these enzymes in the conjunctiva may offer a means to limit the disease.

## The impact of Trichiasis and Trichiasis Surgery

Trichiasis results in significant morbidity due to the visual impairment and pain that it causes. As part of the estimates of the Global Burden of Disease, trachoma related vision impairment is included in the calculations.^31^ Previously, there have been attempts to estimate the morbidity attributable to trichiasis. However, there is relatively little data to base such calculations on.^32^ Moreover, we know relatively little about the impact that this condition has on the lives of people with trichiasis or the effect that surgical treatment has on the quality of life, function and wider issues such as poverty.

Clinical measures outcomes do not provide the full picture of the impact of a condition on the life of the individuals and their families. In eye care the most common measure of the impact of a disease or an intervention to treat it is visual acuity. However, for patients the most important thing is usually not whether their vision is 6/18 or 6/6 following a certain intervention, but rather, what the impact it has on their overall functioning in life. Trichiasis can have a profound effect on many aspects of the individual life. In this study, we aim to measure the impact of un-operated trichiasis and then subsequently of trichiasis surgery on the quality of life, activity, time use and economic welfare of the patient using various tools.

#### Quality of life

Quality of life refers to the general well-being of the individual. It can be measured quantitatively with varied contexts. Tools used to measure quality of life can be classified in to: health related quality of life (HRQoL) tools and tools measuring broader concept which can evaluate the overall experience of life. The HRQoL tools can further be divided into tools measuring disease specific quality of life and generic HRQoL.

#### Vision related quality of life (VRQoL)

The disease specific quality of life tools range from those measuring the functional ability of patients such as restriction in daily activity in relation to specific disease to the impact of a condition to a broader area of health such as psychological and social aspects. Few studies have used the disease specific tools to measure functional ability of trichiasis patients before and after surgery. A population-based study in Tanzania identified that the trichiasis alone results in a limitation comparable with visual impairment on physical functioning in women^33^. In Ethiopia, trichiasis caused similar level of disability to cataract.^34^ In another study the physical functioning of trichiasis cases found to be improved 6 month after undergoing surgical treatment. ^35^ A comprehensive vision related quality of life (VRQoL) measure is recently available (WHO/PBD VFQ). This tool measures the impact of visual impairment in the person’s life including mental wellbeing, dependency and social functioning.

#### Generic HRQoL

Generic HRQoL tools are capable of assessing a range of health concepts and can be used irrespective of specific disease or condition. Most of the time these tools are comprehensive and cover multiple domains of health from the individual’s perceptions of his/her own health to social and environmental functioning. The WHOQOL-BREF (WHOQOL Group, 1998) has good applicability in low and middle-income countries (LMIC) as it was developed simultaneously from concept across 18 countries including LMIC in Africa, Asia and Latin America. It measures 4 domains of health: Physical Health, Psychological Health, Social Relationships, and Environment.

No adequate studies have been conducted to assess the impact of trichiasis and trichiasis surgery on quality of life of patients. One hospital based prospective interventional study conducted in India has compared the quality of life of trichiasis patients and patients with entropion with age, sex and socio-economic status matched patients without trichiasis using the WHOQOL-BREF tool. The study showed that trichiasis patients have poorer quality of life than the non-trichiatic patients in the physical and psychological domain regardless of the presence of visual impairment prior to intervention (epilation for minor trichiasis and surgery for entropion and major trichiasis). Two to four weeks after the intervention, quality of life scores improved regardless of visual improvement.

However, this Indian study had a number of significant limitations. Firstly, about two third of the “trichiasis cases” had no trichiasis (only entropion), which precludes drawing conclusions about the change in the quality of life of trichiasis patients. Secondly, the study used hospital control many of whom had other potentially impairing conditions which might influence their quality of life scores.

#### Trichiasis Pain

Trichiasis can cause quite marked pain. However, no previous study has explored this issue in depth. It is important to have a better understanding that this has on the life and well-being of people with trichiasis. A few studies have asked a simple question about the effect of surgery on the pain associated with trichiasis, however, this is another aspect that needs to be investigated in more detail.^26^^,^^36^^,^^37^

#### Activity and Time Use

Anecdotally, trichiasis can interfere with many activities such as daily personal and household activities, work activities and leisure/social activities. However, no data is available to support this assumption. The ‘stylised activity list’ developed for the World Bank’s Living Standards Measurement Survey (LSMS) allows to assess various activities and the amount of time allocated for each activity.^38^

#### Trachoma and Poverty

Poverty and trachoma are highly correlated. Trachoma overwhelmingly affects poor and marginalised communities, predominantly in Africa and Asia. Poor communities often have: limited access to clean water and sanitation and crowded living conditions. These factors have been repeatedly associated with trachoma, probably because they promote the spread of *C. trachomatis* infection.

Blindness or visual impairment from trachoma is likely to lead to a loss of income at the individual level, which may contribute to family or individual financial poverty. The burden of disability caused by trachoma was estimated at 1.3 million DALYs, with economic losses of 5-8 billion USD/year.^39^^,^^40^ However, the effect of trachoma on income may begin prior to the visual impairment. The pain and photophobia from trichiasis alone are disabling, and may exacerbate the pre-existing financial strain / poverty. Previously healthy productive adults can be rendered dependent on others and unable to work or fully care for themselves due to the trichiasis, compounding poverty. A study in Tanzania, showed that women with trichiasis but without visual impairment had a comparable degree of disability to those with visual impairment. The economic loss from trichiasis alone due to lost productivity was estimated to be about three billion USD per year.^39^^,^^40^ However, these relatively old economic estimates are based on very limited data. There is a need for more up-to-date and detailed measures of the economic cost of the disease.

In this study, we aim to address this gap and we will compare the economic welfare of trichiasis patients with their non-trichiatic counterparts and investigate if trichiasis surgery actually improves the economic welfare of trichiasis patients.

# Rationale

The two commonest trichiasis surgical procedures (BLTR and PLTR) have not been compared in an adequately sized trial with sufficient follow-up time. The important question of which of these two commonly used alternatives gives the best results under programmatic conditions remains unanswered.

Therefore, there is a need to rigorously examine with an appropriate sample size and an adequate follow-up period in a programmatic setting, which of the two operations (BLTR or PLTR) performs better. This question was identified as a research priority several years ago by the WHO/GET2020 meeting.^25^ If one of these procedures does indeed turn out to have better results, this would have major implications for how the surgery is performed and results will be fed directly to guide policy.

There have been no adequate studies which have measured the impact of trichiasis and trichiasis surgery on vision and health related quality of life, activity and household poverty. The impact of trichiasis pain on the individual’s functional health and well-being has never been studied. This study will fill this knowledge gap by measuring the impact of the trichiasis surgery on the life and wellbeing of individuals. The data will help programme planners and funders to secure resources for prompt trachoma prevention and trichiasis intervention.

# Study Objectives

*Primary Objective*:

To determine whether the Bilamellar Tarsal Rotation to Posterior Lamellar Tarsal Rotation has the lower recurrence rate under operational conditions in patients with trachomatous trichiasis at 6 and 12 months after surgery.

*Secondary Objectives:*

- To determine whether there is difference in the complication rates between the two procedures.
- To determine whether there are differences in changes of corneal opacification or visual acuity at 6 and 12 months postoperatively.
- To investigate risk factors contributing to recurrence in patients who have received trichiasis surgery.
- To measure the impact of trichiasis surgery on quality of life, activity, pain and household poverty.

# Four-year follow-up study objectives

Primary objective:

Determine if the results of PLTR and BLTR surgical procedures are different 4 years after surgery and give different results from what has been found at 1 year.

Secondary Objectives:

- Determine if eyelid contour abnormality (ECA) resolve or regress through time
- Determine factors influencing long-term outcomes in PLTR and BLTR surgery
- Evaluate vision and corneal opacity changes four year after trichiasis surgery
- Measure mental health status of people surgically treated for trachomatous trichiasis
- Measure the impact of poor surgical outcomes on mental health

# Methods

## Study Design

In this prospective double-masked, randomised clinical trial (RCT) we will investigate which of the two commonly used alternative surgical procedures for the treatment of trachomatous trichiasis provides the better result in terms of reduced recurrence. Individuals with un-operated trachomatous trichiasis will be randomised to one of the following two intervention arms:

Bilamellar tarsal rotation surgery (BLTR), performed under local anaesthesia

Posterior lamellar tarsal rotation surgery (PLTR), performed under local anaesthesia

Currently both of these operations are widely used in Ethiopia and are approved for the treatment of TT by the WHO.

We will investigate the impact that trichiasis surgery has on a change in the quality of life, activity and economic welfare between trichiasis patients and unaffected individuals (“unexposed controls”) at baseline and 1 year after surgery, a cohort study will be nested within this RCT.

## Study Area and Population

This study will be carried out in Amhara Region, Ethiopia. Amhara Region has the highest burden of trachoma among the 9 regional states of Ethiopia. The region is sub-divided into 11 zones; each containing a population or one to two million people. The western part of Amhara has the highest burden of trachoma, with a trichiasis prevalence ranging between 5 and 10%. According to a report from The Carter Center Ethiopia in 2011, five zones in the west Amhara together contribute to 58% of the regional trichiasis backlog. There is a well established trachoma control programme in this area.

It is anticipated that the study participants (individuals with un-operated trichiasis) will be recruited from multiple woredas in West Gojam zone (such as Mecha Woreda, South and North Achefer Wordas, Yilaman Densa Woreda, Bahirdar Zuria Woreda, Gonji Kolelal Woreda, Burie Woreda, Sekela Woreda, Dembecha woreda, Jabitenan woreda...etc) through community-based identification and by organising community-based surgical outreach campaigns in various health facilities. However, the specific number and exact location of recruitment sites within these woredas is not fixed at this point as it will be influenced by where the people who need the surgery services are actually living, the topography of the woredas as well as the participant flow during the recruitment months. Based on recent prevalence data, it should be possible to recruit 1000 trichiasis patients in a single woreda in West Gojam Zone. However, all villages in these woredas are not accessible by car. For the sake of the follow-up, woredas where most of their villages are accessible by car will be used for the recruitment. If, for instance, we find the required 1000 persons in Mecha Woreda in West Gojam zone, the recruitment will be limited and conducted in this woreda only. Otherwise, the recruitment will continue to other sites and woredas of west Gojam zone and even to the adjacent woredas of East Gojam and South Gondar zones. The majority people in these zones are of Amharan ethnicity and have similar life style and traditions providing a comparable study population.

## Research Partnership

This study will be conducted through a collaborative research partnership between the Amhara Regional Health Bureau, the Carter Center trachoma control programme and the London School of Hygiene and Tropical Medicine. These three organisations have worked together on trichiasis surgery research since 2007, and have a strong track record. The study coordination office is located in Bahirdar, Our research team has extensive experience of working in this area.

## Sample Size

From the STAR trial, the one-year TT recurrence rate using the BLTR was about 10% (tetracycline group).^41^In our recent trials in Ethiopia involving patients with a similar severity of disease to the STAR trial, we found PLTR had a one-year recurrence rate of 18%.^27^ We estimate we will need a sample of 836 to detect a reduction in TT recurrence from 18% to 10%, with 90% power and 95% confidence. Therefore, we would aim to recruit 1000 (500 in each arm), to allow for about 15% loss to follow-up.

For the trichiasis impact study, in parallel with the above 1000 trichiasis cases, we will recruit 200 people without trichiasis (“healthy”/unexposed controls) who are location, sex and age (within 5 years interval) matched with every fifth trichiasis case (which are called from now on comparison cases). The data from these “unexposed healthy controls” will be used to make comparison of changes in quality of life, activity and economic welfare with the trichiasis patients at baseline and 1 year following intervention. The sample size is calculated with the aim of detecting a 10 point difference in Per Capita Expenditure (PCE) between exposed (trichiasis cases) and unexposed (healthy controls) one year after enrolment. Accordingly, 200 trichiasis cases and 200 unexposed controls would provide a 90% power to detect moderate effect of trichiasis surgery on PCE with an effect size of about 0.33 (effect size = PCE difference/SD). .

## Trichiasis cases

#### Case Definition

Trachomatous Trichiasis cases in this study will be defined as: individuals with one or more eyelashes touching the eyeball or with evidence of epilation in either or both of the eyes, and tarsal conjunctival scarring.

#### Inclusion criteria

1. Trachomatous Trichiasis with tarsal conjunctival scarring
2. Adults (≥18 years)
3. Agree to be randomly allocated to one of the two alternative operative procedures
4. Agree to re-examination at six and twelve months after surgery

#### Exclusion criteria:

1. Age less than 18 years
2. Previous eyelid surgery
3. Medically unfit, BP systolic >170, diastolic >110.
4. Pregnant
5. Patients with trichiasis from other eyelid pathologies, such as:
   - Blepharitis
   - Steven Johnson syndrome
   - Burn
   - Trauma
   - Eyelid muscle sub-laxity due to aging
   - Herpes zoster
   - Ocular cicatricial pemphigoid

#### Recruitment

Recruitment of trichiasis cases will be done through community-based identification and by organising community-based surgical outreach campaigns (this is the current established practice in this part of Ethiopia). A mobile eye care team will systematically visit communities in the districts, as per the programmes standard practice. People with trichiasis will be identified through a number of means such as self-presentation after a community mobilization, door-to-door screening of older members of the community or identified by the local health worker. The recruitment is achievable within about a six-month period. We have recently completed two RCTs on trichiasis management in this area of Ethiopia; the recruitment period for 2600 study participants took 4 months.

#### Triaging procedure

The following triaging procedure will be used during the surgical outreach campaigns:

a. All people presenting at the recruitment location will be examined using magnifying loupes and a torch for trichiasis. To check for exclusion criteria they will be asked their ages and history of previous surgery. If they are a woman of childbearing age, they will also be asked whether they might be pregnant.
b. ***Patients with trachomatous trichiasis and no exclusion criteria:*** will be invited to enrol in the study. The study information sheet will be read to them in Amharic. If they are willing they will then be taken through the informed consent process (Appendix 3).
c. ***Patients with trachomatous trichiasis and an exclusion criterion:*** will not enter into the study, but will be offered appropriate standard treatment (Epilation or Surgery).
d. ***Patients with non-trachomatous eye disease:*** will not enter this trial, they will be examined during the recruitment day:
   i. Those with trachomatous eye disease but without trichiasis will be offered standard treatment
   ii. Non-emergency conditions: manageable condition at field setup will be provided treatment. Otherwise a clear instruction to attend the nearest eye unit with a referral letter will be provided.
   iii. Emergency conditions: will be referred immediately to the nearest eye health facility or hospital
e. ***Trichiasis patients refusing surgery:***

These will be counselled to undergo surgery. If they still refuse, they will be offered epilation forceps with clear instruction on how to do the epilation. They will be re-examined a year after to evaluate the course of their condition.

## Unexposed/Controls

Unexposed/controls in this study are defined as: individuals without trichiasis or history of trichiasis or evidence of epilation who are location, sex and age (within 5 years interval) matched to the corresponding cases.

One unexposed person/control will be recruited for every fifth TT case. The study team will visit the home village of each of the cases selected to participate in this cohort part of the study. With the assistance of the case, they will identify potential control subjects.

#### Unexposed/controls recruitment procedure

1. The comparison case and his/her household head will be asked to lead the team to the nearest neighbour from a different family who is sex and age matched with them
2. If there is more than one person meeting the eligibility criteria, one will be chosen randomly (using lottery method).
3. If suitable individuals can be identified as potential control participants from the case’s immediate neighbours, the village administrator will be asked to assist in finding a a suitable control in the wider local community.
4. If eligible controls from the village (“Got”) are not found, recruitment will be done in the next nearest village
5. Individuals who are eligible and willing to participate will be enrolled into the study after informed consent
6. Individuals with identified vision impairing conditions such as cataract and trichiasis will be referred for appropriate treatment

#### Unexposed/controls eligibility criteria

1. Same sex as the case being compared
2. Age +/- 5 years
3. No trichiasis or history of trichiasis
4. Living in the same village / community
5. Willing to participate in this study

## Informed Consent

Participants will be informed about the nature of the study and asked if they would be willing to participate. Cases will be asked if they would be willing to be randomised to alternative surgical procedures and be followed for a year. The information document will be read to them in Amharic. Close-up photographs of the eyelids and cornea will be taken, from which it would not be possible to recognise the individual. In addition a photograph of the participants face will be taken at baseline to help with the follow-up. This will be documented and stored separately from the clinical record forms to avoid breach of confidentiality. These are specifically listed on the information and consent form (Attached).

Information on household expenditure, asset and self rated wealth will be collected after an informed verbal consent is given by the household head in addition to the informed written consent by the study participant.

Enrolment into the study will be dependent on witnessed informed consent, which will be recorded on a consent form marked by the participant’s signature or thumbprint. Those trichiasis patients who decline enrolment in the study would still be offered surgery by the team. The informed consent process will be conducted by an experienced and well-trained field worker; they will be supported by an ophthalmic officer trained in eyelid surgery and the details of the study, and therefore competent to provide answers to questions. The detailed procedure for taking informed consent is described in Appendix 1.

## Baseline Assessment

#### Questionnaire

At the beginning, a brief demographic questionnaire will be used to record name, gender, age, address, ethnicity and literacy of all participants (Attached). A brief pre-operative ophthalmic questionnaire will be used to elicit information on the duration of trichiasis, history of epilation, pain from trichiasis and other trichiasis related concerns for TT cases only. Field workers using the following tools will interview both trichiasis cases and the controls.

#### Vision related quality of life (VRQoL) tool (WHO/PBD VFQ(20))

This is a vision specific quality of life questionnaire developed by the WHO as a cross-cultural tool for assessing vision in low-income settings. This tool has recently been validated in field testing in Kenya and showed good psychometric properties in assessing vision related quality of life associated with cataract visual impairment.^42^ This instrument consists of 20 questions divided into three sub-scales: visual symptom, general functioning and psychosocial. It begins by asking the patient *“Overall, how would you rate your eyesight using both eyes?”*; and uses a five point scale answer option such as *“very good”, “good”, “moderate”, “bad”, “very bad”* (See study record form). The test is scored out of 100, with higher scores reflecting a better VRQoL.

#### WHOQOL-BREF

The WHOQOL-BREF is a shortened and comprehensive version of the original 100 item WHOQOL instrument, producing 4 subscales on Physical Health, Psychological Health, Social Relationships, and Environment (See study record form). This is a useful measure of QoL in low and middle income countries and is adapted in Ethiopia. It asks respondents 26 questions how much (frequency) they have experienced and/or were able to do things (e.g. feel safe, able to concentrate, enjoy life) in the past 4 weeks and how satisfied they are with certain aspects of their lives (e.g. sleep, capacity for work) ^43^.

#### Activity and Time Use tool

Activity and time use data will be collected using a modified ‘stylised activity list’ developed for the World Bank’s Living Standards Measurement Survey (LSMS).^38^ Participants will be asked whether they had been involved in each of a list of common daily activities during the last week. If they were involved, they will be asked how much of their time was used for each particular activity. The tool will contain following main components:

a. **Participation:** This will explore whether or not the person had undertaken the following activities during the previous week:
   - Personal activities: sleep, rest, self care, etc.
   - Household/family activities: cooking, shopping, house cleaning/maintenance, child care, etc.
   - Work/Job (productive activities): Paid work, agriculture, animal husbandry, selling, fetching water, etc.
   - Social and community activities: social reunions, leisure activity, visiting, etc.
b. **Time:** the amount of time spent on different activities during the previous “working” day
c. **Assistance:** whether or not the person had assistance during the previous week while doing any of the above activities

#### Tools measuring household poverty

Poverty is a complex multidimensional problem with various causes and manifestations.^44^ Accordingly, there are various methods of measuring poverty. These include financial measures such as measuring income, household expenditure, assets and self/peer rated wealth; nutritional measures such as measuring food insecurity, nutritional status and calorie consumption; non-monetary measures such as assessing occupation, education and literacy. In low-income countries poverty should be measured at the household level rather than of the individual level as resources are shared within the household. In this study we will use the following methods to measure household poverty.

a. ***House hold per capita expenditure:***

This will be assessed using a modified setting specific household expenditure measurement tool developed for the World Bank’s Living Standards Measurement Survey.^38^ The person responsible for finances in the household will be interviewed to assess household consumption during the previous month of goods produced through home-production, bought, or received as gifts or payment. Items on food, education, household expenses and personal expenses and rent will be included (Appendix 4). The value of these items will be summed and divided by the number of household members to calculate household monthly per capita expenditure.

b. ***Asset:***

Data will be collected on standard indicators of socio-economic-status including number and type of different assets (e.g. cattle, furniture, electrical goods) and household characteristics (e.g. building materials of the floor, roof and walls; type of toilet and number of rooms) using a local context specific questionnaire (Appendix 4).

c. ***Peer rated wealth:***

Three member of village administration living in the same village (“Got”) with the cases and the controls will be randomly selected and will be asked the following question:

*“How well-off do you think [name of household head] household is in relation to the other households in the village?”* Then they will be asked to rate the household from the options: *“very poor”, “Poor”, “Average: Neither Poor nor Wealthy”, “Wealthy” and “Very Wealthy”* (Appendix 4). The responses will be given weight and will be averaged to produce a score of the household’s level of wealth in relative to the other households in the village.

#### Clinical examination

Near and distance visual acuity and contrast sensitivity will be measured. A single observer will examine the participants’ eyes with magnifying loupes and a torch for signs of trachoma using the detailed WHO grading system, with additional assessment of entropion and trichiasis severity (Appendix 2).^45^ Digital photographs will be taken of eyelid and cornea for independent grading. A photograph of their face will also be taken to help confirm their identity at follow-up. Once the examination is completed the patient will be guided to a separate area where the surgical procedure will be randomly allocated. Please refer to the detailed clinical examination protocol in Appendix 2.

## Randomisation, Allocation Concealment and Masking

#### Randomisation

Immediately after the baseline examination, patients will move to a separate area where the “randomisation administrator” will conduct the randomisation process. Patients will be randomised into either of the treatment arms: BLTR Surgery or PLTR Surgery. There will be about 6 surgeons performing surgery during the trial, although only 2 or 3 working on any given recruitment day. The patient will be allocated to the next available surgeon. As surgical recurrence rates can vary between surgeons, each surgeon will have their own randomisation sequence. The randomisation administrator will inform the surgeon which operation is required. In bilateral cases, both eyes will receive the same operation. However, only one eye will be included in the primary analysis. The “trial eye” in bilateral cases will be chosen at random. The statistician will prepare a random list (right or left) to determine which eye will be the study eye.

The following particular randomisation procedure will be followed:

a. Randomisation administrator will be working in a location not visible to other project team members.
b. After examination, the patient will be sat in a waiting area.
c. When a surgeon becomes available to operate, the randomisation administrator and the surgeon together take the patient through to the theatre.
d. Again, together they take the next envelope from that surgeon’s sequence and open it. The envelope will contain a piece of paper printed with the randomisation number and the allocated surgical procedure (either PLTR or BLTR) for this number, with the same randomisation sequence number on the outside.
e. They will then record the allocation on the randomisation allocation masterlist (in duplicate). One copy will be kept by the randomisation administrator and the second copy being given to a person independent of the trial for safe keeping and independent data entry. A new masterlist page will be started on each trial day.
f. The randomization administrator will ask the surgeon to double check that the correct procedure is going to be used. They will both sign on the masterlist to confirm that correct surgical procedure is used
g. The surgeon will record the details of the operation in a logbook, to record the procedure type and any surgical complications.

#### Allocation Concealment

The randomised allocation will be concealed in opaque, sequentially numbered envelopes. The sequences will be prepared by the trial statistician, who will securely hold the master list of the allocated treatment. To ensure reasonable balance in the allocation of treatments in different locations, the sequences will be blocked into groups. The size of the blocks will be determined by the statistician and not divulged to the study team.

#### Masking

Both the trial participant and the observer will be masked to the intervention arm until the end of the trial. Unmasking is not likely to be applicable as the treatment is not ongoing. Once the surgery is performed it is not possible to identify the type of surgical procedure used for treatment.

## Trichiasis Surgeon Standardization

The Amhara Region trachoma control programme, which is supported by The Carter Center has trained more than 1000 trichiasis surgeons on the PLTR surgical procedure in the last 10 years. Among these surgeons, eight certified and highly productive trichiasis surgeons from West Gojam Zone will be selected, trained and certified in the WHO recommended BLTR trichiasis surgical procedure by an experienced Ethiopian Ophthalmologist. From among these eight surgeons, those who have performed at least 50 trichiasis surgeries using each surgical procedure will be reassessed and standardized by a second Ethiopian ophthalmologist, according to the procedures described in the WHO manual for “The Final Assessment of Trichiasis Surgeons”. It is expected that six surgeons who are certified to perform both the BLTR and the PLTR operations will finally be chosen to perform the surgeries for this trial.

## Surgical Treatment

The randomised treatments used in this trial will be one of the WHO approved surgical procedures for the treatment of trichiasis: BLTR or PLTR. The surgery will be performed in health centres and will be provided to the participants free of charge. The trichiasis operation takes about 15 minutes. All procedures will follow strict aseptic technique. The patient needs to lie flat while the surgery is done.

First, the eyelid and the surrounding area will be cleaned. The face will be covered with a sterile towel with appropriate opening to access the eyelid. The conjunctiva is anaesthetised with a drop of topical anaesthetic (proxymetacaine or tetracaine). The conjunctival sac will be cleaned using a drop of topical povidone iodine 5% ophthalmic solution. Then local anaesthetic (lidocaine 2% with adrenaline [1:100,000] injection) will be injected into the skin of the eyelid to prevent pain during the surgery. After surgery, tetracycline eye ointment will be applied on the sutured lid prior to patching it for about 12 hours.

All patients will be provided with 1% tetracycline eye ointment produced by a single manufacturer, Galentic Pharma Pvt. Ltd., India (This is what is currently supplied by Pharmaceutical Fund and Supply Agency, Bahirdar Branch) with instructions of how to apply it twice a day for two weeks. The trichiasis surgeons and the surgical assistants will demonstrate to all patients and/or their relatives the amount of the tetracycline ointment to be applied and the part of the eyelid it shall be applied.

Being a randomised trial the random allocation of patients to either of the intervention arms ensures no systematic difference or bias between intervention groups in both known and unknown factors that may affect outcome. Accordingly, it is expected that any differences in dosage frequency and compliance of the tetracycline eye ointment across the two arms will be equal or balanced due to the random allocation and is unlikely to introduce systematic bias or influence outcome. In addition, the analysis will be done based on intention to treat; irrespective of the trial participants’ compliance to the intended intervention. Analgesics (paracetamol) will be provided as needed. All patients will be told when to come back to the next follow-up date.

#### Treatment arms

#### Arm A: BLTR surgical procedure

In the BLTR surgical procedure firstly an incision is made through the skin and orbicularis oculi muscle parallel to and 3mm above the lid margin. This is followed by evertion of the lid and a second incision through the conjunctiva and tarsal plate. Then the incisions are united. At this point the eyelid should be divided through its entire thickness, 3 mm from and parallel to the lid margin, remaining connected at both ends. Then the lid will be rotated and sutured with three everting sutures. Please refer Appendix 3 for the detailed procedure.

Figure 1: **Bilamellar tarsal rotation:** sagittal view of the upper eyelid (a) Bilamellar incision through the eyelid. (b) Horizontal mattress suture. (c) Post-operative lid eversion with suture tightening.

#### Arm B: PLTR surgical procedure

In the PLTR surgical procedure, after everting the lid, an incision is made in the tarsal conjunctiva through the tarsal plate until the orbicularis muscle is reached. Then the tarso-conjuctiva is blunt dissected from the orbicularis muscle. Then the lid will be repositioned and sutured with three everting sutures. Please refer Appendix 4 for the detailed procedure.

Figure 2: **Posterior lamellar tarsal rotation:** sagittal view of the upper eyelid (a) Posterior lamellar incision. (b) Dividing anterior and posterior lamellae. (c) Horizontal mattress sutures. (d) Post-operative lid eversion.

#### Intra-operative and post-operative examinations

During the operation, an experienced ophthalmic officer will examine and document the incision length, suture position, duration of surgery and complications. The operated lid will be examined for degree of correction, eyelid contour abnormality and lid closure immediately after the completion of the surgery (Appendix 4). A photograph will be taken of the eyelid position at the end of the operation for independent grading.

## Follow-up Assessments

All individuals with trichiasis who are enrolled into the RCT and operated for their trichiasis will be re-assessed at three time points at 7-10 days, 6 months and 12 months from the point of enrolment. All participants will be given appointment cards for their next follow-up and will be reminded about the follow-up a week prior to the date. The “village development army” and the 1 to 5 village organization system will be used to locate the subject’s home to aid the follow-up process.

The “healthy controls”, enrolled into the impact evaluation case-control study, will only be re-assessed at 12 months after enrolment.

Participants who do not present for their follow-up visit will be traced. Reasons for the loss to follow-up will be identified. If sick or moved within accessible areas, a follow-up assessment will be arranged in their houses or villages. The number of participants who are not traceable will be recorded and included in the monthly report to the Amhara Regional Health Bureau.

#### Questionnaire

At 7-10 days after surgery, a brief ophthalmic questionnaire will be used to ask patients for any discomfort after the surgery and their impression of the surgery they have received.

At 6 months a brief ophthalmic questionnaire will be used to ask patients current symptoms and their impression of the surgical treatment they have received.

At 12 months a brief ophthalmic questionnaire and all other tools used at baseline will be used to evaluate the impact of trichiasis surgery on pain, productive activity and participation, quality of life and household poverty in trichiasis patients (cases). Controls will be re-interviewed at 12 months using the same tools used during the baseline recruitment and their visual acuity and contrast sensitivity will be re-measured.

#### Clinical examination

For cases, the visual function tests (near and distance visual acuity and contrast sensitivity) will be re-measured at 6 and 12 months. On each of the three follow-up occasions the eyes will be re-examined for signs of trachoma and recurrence, which will be graded using the detailed WHO trachoma grading system. Photographs will be taken of the eyelids and cornea.

For controls, the visual function tests (near and distance visual acuity and contrast sensitivity) will be re-measured at 12 months.

Any patients with recurrent trichiasis will be offered surgical treatment or epilation. Participants with other ocular conditions will be advised and referred to the nearest eye health facility.

## Fourth Year Follow-up Assessments

All trichiasis cases that were enrolled into the RCT and all non-trichiasis controls enrolled in the nested trichiasis surgery impact study would be followed at four years after enrolment. The “Eye Ambassadors” used for case finding at baseline and the 1 to 5 village organization system will be used to locate the subject’s home to aid the follow-up process. Participants who do not present for their follow-up visit will be traced. Reasons for the loss to follow-up will be identified. If sick or moved within accessible areas, a follow-up assessment will be arranged in their houses or villages. The number of participants who are not traceable will be recorded and included in the monthly report to the Amhara Regional Health Bureau.

### Informed Consent

Participants will be briefed about the aim of the four year follow-up. All participants will be asked if they would be willing to participate in the four year follow-up study. The information document will be read to them in Amharic. Cases will be clearly told that close-up photographs of the eyelids and cornea will be taken, from which it would not be possible to recognise the individual. In addition, a photograph of the participants face will be taken. This will be stored separately from the clinical record forms to avoid breach of confidentiality.

Enrolment into the four year follow-up study will be dependent on witnessed informed consent, which will be recorded on a consent form marked by the participant’s signature or thumbprint. Trichiasis patients who decline participation in this study but who had recurrent trichiasis would still be offered surgery by the team. Experienced and well-trained field workers will conduct the informed consent process.

### Questionnaire

A brief ophthalmic questionnaire will be administered. The mental health status of the trichiasis cases will be assessed using two mental health assessment tools (Patient Health Questionnaire - 9 (PHQ – 9), and Self Reporting Questionnaire (SRQ20)), and other qualitative methods such as in-depth interviews and focus group discussions. Controls will be re-interviewed using the same quantitative and qualitative mental health assessment tools for comparison of results with the cases.

### Clinical examination

Visual function tests will be done in both cases and controls. For cases, eyes will be re-examined for signs of trachoma and recurrence and other surgical outcomes, which will be graded using the detailed WHO trachoma grading system using 2·5× binocular loupes and torch. Photographs will be taken of the eyelids and cornea. Any patients with recurrent trichiasis will be offered surgical treatment or epilation. Participants with other ocular conditions will be advised and referred to the nearest eye health facility.

## Data Analysis

#### Data management

Data will be archived in the locked, dedicated study office, which is located within the secure compound of the Carter Center programme office in Bahirdar. Paper data will be filed based on registration number and can only be accessed by the trial coordinators. Picture data will only be collected and stored in electronic copy in a password protected computer. The data will be double entered in to Microsoft Access and stored in a password protected computer isolated from the internet in this secured office. Participants’ identification data is only required for the registration and follow-up process. Analysis will be done using an annonymised dataset.

#### Analysis of primary outcome measures

*Intention to treat analysis of risk factors for recurrence*: It is expected that the important baseline characteristics (those that are known to affect the risk of recurrence) will be balanced between the two arms by randomisation (stratified for operating surgeon). If this is found to be the case, the outcome of the surgery in the two arms will be compared in an unadjusted logistic regression model for recurrent trichiasis at one year. If the arms are found to be substantially imbalanced with respect to, pre-operative severity, gender or age, an appropriately adjusted logistic regression model will be used. Individuals will be considered to be recurrent trichiasis cases if they have developed recurrent trichiasis at anytime during the one-year follow-up period. Interim analysis is not planned.

#### Secondary analysis

*Effect modification:* We will assess effect modification of the intervention on recurrence at one year with the following factors by including an interaction term with treatment arm in the logistic regression model.

- Operating surgeon
- Pre-operative severity
- Sex
- Age group
- Conjunctival (papillary) inflammation
- Surgical complications

*Analysis of risk factors for recurrence:* A multivariate logistic regression model will be used to identify potential explanatory factors for recurrent trichiasis at one year, in addition to the randomised surgical procedure type allocation.

*Impact of intervention on time-to-first-recurrence:* Kaplan-Meier analysis will be used to plot the survival curves for both treatment arms up to the final visit at one year. Cox regression will be used to assess the impact of the intervention on time to first recurrence. The hazard ratio will be estimated with Cox regression, adjusting for substantial baseline imbalances if appropriate.

#### Impact analysis

Analysis of the health related and vision related quality of life data will be done based on the specific scoring and analysis procedures provided by the WHO. The raw scores will be calculated and converted to a scale of 0 – 100. Higher score will represent a best score. The relationship between QoL, visual acuity and socio-demographic variables will be analysed using Analysis of Variance (ANOVA) and chi square tests. The pain impact score will be analysed by summing the weights assigned to the item response options selected by the patient. Higher scores indicate higher pain severity and pain impact on a person’s life.

For the household poverty analysis, the socio-demographic characteristics of cases and controls will be compared using t-test for continuous variables and chi-square for discrete variables. Logistic regression analysis will be undertaken to evaluate the association between the case/control status, socio-demographic and poverty variables. Difference in household poverty level between cases and controls will be assessed using the t-test and the Mann-Witney test for the three poverty variables: asset, expenditure and peer rated wealth. Changes in household poverty overtime will be calculated using the paired t-test and Wilcoxon signed rank test.

Multivariable logistic regression will be employed to compare participation of cases and controls in different group of activities and to investigate predictors for change in productive and in non-productive activities. In addition, participation in activities by level of visual acuity among cases and controls will be assessed by logistic regression analysis and odds ratios and 95% confidence intervals will be presented. Proportion of time spent on different activities between cases and controls will be shown by means and 95% confidence intervals from paired t-tests. McNemars test will be used to compare engagement in activities during the last week (binary variable) between cases and controls.

Data and all appropriate documentation will be stored for a minimum of 7 years after the completion of the study, including the follow-up period.

## Clinical outcomes at four years

The primary outcome will be rate of postoperative trachomatous trichiasis (PTT) at four years,

Secondary outcomes include:

- Rate and regression of Eyelid Contour Abnormality (ECA)
- Visual acuity changes
- Corneal opacity changes
- Factors influencing long-term outcomes in trichiasis surgery

## Fourth year follow-up clinical data analysis

Data were double-entered Access (Microsoft) and transferred to Stata 11 (StataCorp) for analysis.

The primary outcome and binary secondary outcomes such as eyelid contour abnormality (ECA) will be compared between the two surgical arms with logistic regression analyses to estimate the odds ratio (OR) and 95% confidence interval (CI). All comparisons between the two surgical procedures will be controlled for surgeon effect as a fixed effect in the model. The risk ratio (RR) and risk difference in the primary outcome (recurrent trichiasis at four year) between BLTR and PLTR procedures will be analysed. The possibility of effect modification between arm and a-priori defined factors such as surgeon, preoperative trichiasis severity, papillary inflammation, age and sex will be investigated. Ordered categorical secondary outcomes will be compared between the two surgical interventions using ordinal logistic regression. Categorical secondary outcomes will be analysed using multinomial logistic regression to estimate relative risk ratio (RRR) and 95% CI. Negative binomial regression will be used to analyse the difference in the number of recurrent lashes touching the eye between the two intervention arms.

## Four-year follow-up mental health data analysis

Quantitative data will be analysed specific procedures designed to analyse data collected using the mental health tools employed. Mental health status scores between cases and non-trichiatic controls will be compared using linear regression method, an t-test will be employed to compare significant differences in mental health scores between cases and controls. Logistic and ordinal logistic regression methods will be used for binary and ordered categorical outcome variable analysis, respectively. All comparisons of cases and controls will be adjusted for the matching variables: age and sex; and for both socio-economic status and presence of health problems during the previous four weeks, as these factors may confound mental health scores in cases and controls.

Mental health scores of cases with postoperative trachomatous trichiasis and moderate to severe ECA at four years will be compared with those with successful surgical outcomes using linear regression model.

For the qualitative data, major themes will be coded and presented. Important views will be directly quoted.

## Study Duration

It is anticipated that the project will take four years to complete. The fieldwork component is expected to take about 2 years. Each subject will be followed-up for 1 year from the time of the surgery. It is likely that the recruitment and surgery phase will take approximately six months.

The four-year follow-up project will take about a year: 5 months field work and 7 months for data analysis and write-up.

# Ethics Approval

This study has been approved by the London School of Hygiene & Tropical Medicine’s Ethics Committee and Emory University IRB. Ethics approval will be sought in Ethiopia from the National Health Research Ethics Review Committee of the Ethiopian Ministry of Science and Technology and Amhara Regional Health Bureau Review Board. The study will begin after approval certificates have been received from these review bodies. The study will be conducted in accordance with the recommendations for physicians involved in research on human subjects adopted by the 18th World Medical Assembly, Helsinki 1964 and later revisions.

Protocol amendment application will be submitted to the London School of Hygiene & Tropical Medicine’s Ethics Committee, Emory University IRB, National Health Research Ethics Review Committee of the Ethiopian Ministry of Science and Technology, and the Ethiopia Food, Medicine and Healthcare Administration and Controls Authority.

# Study Benefits

#### Benefit to the Study Participants

The participants (cases) enrolled in this study will, by definition, have trichiasis. It is recommended by the WHO and the Ethiopian Ministry of Health that people with trichiasis should have lid surgery to correct the trichiasis and thereby preserve their vision. In this study about 1000 trial participants and other trichiasis patients will receive high quality surgical treatment. This is a significant benefit to them, as it reduces the risk of visual loss. Both procedures that will be compared in this trial are approved by the WHO, and are both used widely in Ethiopia and many other countries worldwide for the treatment of trachomatous trichiasis. The surgeries will be performed by properly, certified, experienced trichiasis surgeons who will undergo standardization assessments of their skills using internationally accepted WHO assessment procedures.

Women are approximately three times more frequently affected by trachomatous trichiasis than men, possibly due to a greater lifetime exposure to the infection which promotes more scarring disease. Women may have less access to health care due to cost and transport difficulties. In this study, the surgical service will be provided free of charge in local rural health posts. Preventing the risk of trachomatous blindness in women through high quality surgical treatment means empowering them and improving the living of the general community.

Participants of this study will have a frequent follow-up assessment of their eyes, which currently they are not offered through routine services. The ocular examination will allow identifying other non-trachomatous conditions such as cataract (the commonest cause of blindness in the region) and other external diseases and infections. Those with easily treatable conditions will be managed directly, while others will be advised and referred. Trachoma health education and counselling services, which are an essential component of the SAFE strategy, will be provided to the participants and the community during the publicity phase for the surgical outreach. Medical supervision by an ophthalmologist for this project will be provided through the established structures of Amahra Region eye care programme based in Bahirdar.

About 200 people (controls) without trichiasis will have their near and distance vision tested and undergo ocular examination. This will benefit the participants by the detection of ocular problems. Those with easily treatable conditions will be managed directly, while others will be advised and referred to attend nearest health institutions.

#### General Benefits

The research question this study is setting out to answer was identified as a research priority several years ago by the WHO/GET2020 meeting. The results of this study would have major implications in improving the surgical management of trichiasis and will be fed back to region, national and international programme leaders, to directly guide policy. The impact study will help programme planners and funders to secure resources for prompt trachoma prevention and trichiasis intervention globally.

This is a collaboration project between the Amhara Region Health Bureau, the Carter Center and the London School of Hygiene and Tropical Medicine. Its accomplishment will demonstrate successful partnership in the region, which will attract various health care partnership projects that will greatly benefit the region and its habitants. It is anticipated that participation in this project will provide tremendous work experience to those involved. The capacity of various cadres such as, trichiasis surgeons, regional, zonal and district health officers and primary health workers will be built in the process of implementing the project. In addition, the project will have a job opportunity for more than 10 field workers and for about 3 driver logisticians.

The project will be managed and led in Ethiopia by Esmael Habtamu Ali, who has worked on the joint Carter Center – LSHTM trachoma research project for six years. He is also registered as a PhD student at the LSHTM, and his doctoral thesis will be based on this study. The training and experience that he will receive during the course of this research and his studies will contribute to local research capacity development in eye health in Ethiopia.

# Risk Assessment

This study is considered low risk.

The discomfort or potential risks (discussed below) that participants would be exposed to in this study are no different from those that would be experienced in routine clinical practice in the surgical treatment of trichiasis in Ethiopia. Both procedures are approved by the WHO to be used for the treatment of trachomatous trichiasis and are widely practiced in Ethiopia.^23^ We would expect that the incidence of potential problems to be equal to or less than that seen in routine clinical practice due the greater degree of training, supervision and follow-up in a clinical trial.

The surgery is performed under local anaesthetic, which stings for a few seconds after the injection into the skin of the eyelid. Following eyelid surgery the patient usually has a mild to moderate degree of discomfort in the eyelid for a few days. There is usually some bruising and swelling which settles in about a week. The standard practice is to provide the patient with a short course of simple analgesia, such as paracetamol. We do not anticipate any additional discomfort as a result of using either of the procedures.

The principle problem which can develop following trichiasis surgery is recurrence of the trichiasis. This is the primary outcome in this trial, comparing these two alternative operations. As stated above, the reported rates of trichiasis recurrence range between 10 and 40% at one year. This study will compare alternative procedures to try to identify which has the lowest rate of recurrence. To reduce the risk of recurrence, before starting the study there will be surgical standardisation and assessment of performance of the nurses by a senior Ethiopian ophthalmologist (Dr Amir Bedri), to ensure that all the surgeons are performing the procedure in the same way to a high standard. Day-to-day supervision of the surgical teams will be conducted by an experienced ophthalmic officer and when necessary by an Ethiopian ophthalmologist (Gudie Sharew) based at Bahirdar, who will be responsible for managing complications. If trichiasis does recur it is likely that repeat surgery will be needed. This will be managed under the supervision of the ophthalmic officer and the ophthalmologist.

Infection of the skin can occasionally (less than 1%) develop after surgery and would need to be treated with antibiotics. Careful surgical instrument sterilisation and operative techniques, as well as post-operative topical antibiotic will help minimise this. Sterilisation of equipment will be done using portable steam sterilizers. A log of sterilised material will be kept.

About 7% of people develop a small fleshy lump on the inner surface of the eyelid called a granuloma that can be easily removed.

Bleeding during the operation is a rare problem that can be dealt with at the time by the surgeon.

# Study Monitoring

An independent Data and Safety Monitoring Board (DSMB) will be appointed by the trial steering committee. There will be about 5 members, all independent of the running of the trial with relevant clinical and epidemiological experience.

The DSMB will monitor recruitment and implementation of procedures as per standard protocols. The DSMB may visit the study sites during recruitment and follow up to confirm that patients are treated to a high standard. The DSMB will determine the manner in which it will monitor the data, what it requires from the investigators in this respect and will communicate this to the PIs. It is anticipated that the DSMB will meet at least three times during the recruitment and follow up periods to assess the safety of the trial procedures.

# Study Management

The day-to-day management of the trial will be co-ordinated through the Carter Center, Amhara Region office, in Bahirdar.

The recruitment process will be monitored by senior Ethiopian ophthalmologists (Guadie Sharew and Amir Bedri) for surgical quality.

The fieldwork will be supervised by the trial coordinator (Esmael Habtamu). He will monitor sterilization of equipment, the informed consent and data collection process. He will be the last person to complete the patient data record form, checking for completeness or unusual data values at the recruitment centre before the patient is discharged

***The Trial Steering Committee (TSC):***

Prof Matthew J Burton, LSHTM (LSHTM PI)

Dr Esmael Habtamu, LSHTM (Ethiopia PI and Project manager)

Dr Zerihun Tadesse, Carter Center, Ethiopia

Mr Bizuayehu Gashaw, Amhara Regional Health Bureau

Ms Kelly Callahan, Carter Center, Atlanta

Dr David Macleod, LSHTM

Dr Helen Weiss, LSHTM

Dr Saul Rajak, LSHTM

Prof Matthew Burton is a professor at LSHTM and an ophthalmologist who has spent much of the last 12 years conducting field and laboratory research on trachoma, mostly in The Gambia, Ethiopia and Tanzania. He has been the PI on three previous clinical surgical trials for trachomatous trichiasis, two in the same area of Ethiopia. He is the principal investigator of this trial. He will supervise the overall project.

Dr Esmael Habtamu Ali is public eye health specialist. He has been the study coordinator for clinical trials and has many experience of running four large randomised controlled clinical trials for trichiasis surgery in Amhara region, Ethiopia . He will manage the trial in Bahir Dar, including conducting the clinical examinations and will analyse the data and write the manuscript.

Dr Zerihun Tadesse is the country representative of the Carter Center Ethiopia. He manages the Carter Center trachoma control programme in Ethiopia and has extensive experience in management and running trachoma research projects.

Mr Bizuayehu Gashaw is the Vice Head of Amhara Regional Health Bureau, Bahirdar, Ethiopia. He runs the Amhara Region trachoma control program in Ethiopia and has extensive experience in programme management and research.

Kelly Callahan is the Trachoma Control Programme of the Carter Center, Atlanta. She has been involved in previous randomized controlled trials conducted in Ethiopia. She has extensive research experience in similar settings.

Dr David Macleod, Statistician at the LSHTM is involved in the study design and analysis for this trial.

Prof Helen Weiss, Epidemiologist & Statistician at the LSHTM is involved in the study design and analysis for this trial.

Dr Saul Rajak, Ophthalmologist at LSHTM and previously coordinated two trichiasis surgery trials in Ethiopia.

#### Indemnity

London School of Hygiene & Tropical Medicine holds Public Liability ("negligent harm") and Clinical Trial ("non-negligent harm") insurance policies which apply to this trial.

#### Sponsor

London School of Hygiene & Tropical Medicine will act as the main sponsor for this study. Delegated responsibilities will be assigned locally.

# References

1.	Habtamu E, Wondie T, Aweke S, et al. Posterior lamellar versus bilamellar tarsal rotation surgery for trachomatous trichiasis in Ethiopia: a randomised controlled trial. *The Lancet Global Health* 2016.

2.	Rajak SN, Collin JR, Burton MJ. Trachomatous trichiasis and its management in endemic countries. *Survey of ophthalmology* 2012; **57**(2): 105-35.

3.	World Health Organization Alliance for the Global Elimination of Trachoma by 2020. Second Global Scientific Meeting on Trachomatous Trichiasis. Cape Town, South Africa, 4-6 November 2015.

4.	Resnikoff S, Pascolini D, Etya'ale D, et al. Global data on visual impairment in the year 2002. *Bull World Health Organ* 2004; **82**(11): 844-51.

5.	Mariotti SP, Pascolini D, Rose-Nussbaumer J. Trachoma: global magnitude of a preventable cause of blindness. *BrJ Ophthalmol* 2009; **93**(5): 563-8.

6.	Berhane Y. National Survey on Blindness, Low Vision and Trachoma in Ethiopia: Methods and Study Clusters Profile. *EthiopJHealth Dev* 2007; **21**(3): 185-203.

7.	Emerson PM, Ngondi J, Biru E, et al. Integrating an NTD with One of "The Big Three": Combined Malaria and Trachoma Survey in Amhara Region of Ethiopia. *PLoS Negl Trop Dis* 2008; **2**(3): e197.

8.	World Health O. Future Approaches to Trachoma Control. Report of a Global Scientific Meeting. Geneva, 17-20 June 1996. Geneva: World Health Organization, 1997.

9.	Burton MJ, Kinteh F, Jallow O, et al. A randomised controlled trial of azithromycin following surgery for trachomatous trichiasis in the Gambia. *BrJ Ophthalmol* 2005; **89**(10): 1282-8.

10.	West ES, Munoz B, Imeru A, Alemayehu W, Melese M, West SK. The association between epilation and corneal opacity among eyes with trachomatous trichiasis. *BrJ Ophthalmol* 2006; **90**(2): 171-4.

11.	Rajak SN, Habtamu E, Weiss HA, et al. Epilation for trachomatous trichiasis and the risk of corneal opacification. *Ophthalmology* 2011; **In Press**.

12.	Bowman RJ, Jatta B, Cham B, et al. Natural history of trachomatous scarring in The Gambia: results of a 12-year longitudinal follow-up. *Ophthalmology* 2001; **108**(12): 2219-24.

13.	Burton MJ, Holland MJ, Makalo P, et al. Profound and sustained reduction in Chlamydia trachomatis in The Gambia: a five-year longitudinal study of trachoma endemic communities. *PLoS Negl Trop Dis* 2010; **4**(10): e835.

14.	Burton MJ, Bowman RJ, Faal H, et al. Long term outcome of trichiasis surgery in the Gambia. *BrJ Ophthalmol* 2005; **89**(5): 575-9.

15.	Reacher MH, Taylor HR. The management of trachomatous trichiasis. *RevInt TrachPatholOculTropSubtrop Sante Publique* 1990; **67:233-62.**: 233-62.

16.	Yorston D, Mabey D, Hatt S, Burton M. Interventions for trachoma trichiasis. *CochraneDatabaseSystRev* 2006; **%19;3:CD004008.**: CD004008.

17.	Reacher MH, Huber MJ, Canagaratnam R, Alghassany A. A trial of surgery for trichiasis of the upper lid from trachoma. *BrJ Ophthalmol* 1990; **74**(2): 109-13.

18.	Reacher MH, Munoz B, Alghassany A, Daar AS, Elbualy M, Taylor HR. A controlled trial of surgery for trachomatous trichiasis of the upper lid. *ArchOphthalmol* 1992; **110**(5): 667-74.

19.	Gower EW, West SK, Harding JC, et al. Trachomatous trichiasis clamp vs standard bilamellar tarsal rotation instrumentation for trichiasis surgery: results of a randomized clinical trial. *JAMA ophthalmology* 2013; **131**(3): 294-301.

20.	Rajak SN, Habtamu E, Weiss HA, et al. Absorbable versus silk sutures for surgical treatment of trachomatous trichiasis in Ethiopia: A randomised controlled trial. *PLoS Med* 2011; **8**(12): e1001137.

21.	Rajak SN, Habtamu E, Weiss HA, et al. Surgery versus epilation for the treatment of minor trichiasis in Ethiopia: a randomised controlled non-inferiority trial. *PLoS Med* 2011; **8**(12): e1001136.

22.	West SK, West ES, Alemayehu W, et al. Single-dose azithromycin prevents trichiasis recurrence following surgery: randomized trial in Ethiopia. *ArchOphthalmol* 2006; **124**(3): 309-14.

23.	WHO. Final Assessment of Trichiasis Surgeons. Geneva: World Health Organization, 2005.

24.	Adamu Y, Alemayehu W. A randomized clinical trial of the success rates of bilamellar tarsal rotation and tarsotomy for upper eyelid trachomatous trichiasis. *EthiopMedJ* 2002; **40**(2): 107-14.

25.	World Health Organization. Report of the fifth meeting of the WHO Alliance for the Global Elimination of Blinding Trachoma. Geneva: World Health Organization 2000.

26.	Burton MJ, Kinteh F, Jallow O, et al. A randomised controlled trial of azithromycin following surgery for trachomatous trichiasis in the Gambia. *British Journal of Ophthalmology* 2005; **89**(10): 1282-8.

27.	Rajak SN, Habtamu E, Weiss HA, et al. Absorbable versus silk sutures for surgical treatment of trachomatous trichiasis in Ethiopia: a randomised controlled trial. *PLoS medicine* 2011; **8**(12): e1001137.

28.	Merbs SL, West SK, West ES. Pattern of Recurrence of Trachomatous Trichiasis After Surgery: Surgical technique as an explanation. *Ophthalmology* 2005; **112**(4): 705-9.

29.	Burton MJ, Bailey RL, Jeffries D, et al. Conjunctival expression of matrix metalloproteinase and proinflammatory cytokine genes after trichiasis surgery. *Invest Ophthalmol Vis Sci* 2010; **51**(7): 3583-90.

30.	Burton MJ, Rajak SN, Ramadhani A, et al. Post-operative recurrent trachomatous trichiasis is associated with increased conjunctival expression of S100A7 (psoriasin). *PLoS Negl Trop Dis* 2012; **6**(12): e1985.

31.	Burton MJ, Mabey DCW. The Global Burden of Trachoma: A Review. *PLoS Negl Trop Dis* 2009; **3**(10): e460.

32.	Frick KD, Hanson CL, Jacobson GA. Global burden of trachoma and economics of the disease. *The American journal of tropical medicine and hygiene* 2003; **69**(5 Suppl): 1-10.

33.	Frick KD, Melia BM, Buhrmann RR, West SK. Trichiasis and disability in a trachoma-endemic area of Tanzania. *Archives of ophthalmology* 2001; **119**(12): 1839-44.

34.	Lai H, Melia M, Frick KD, Buhrmann R, West SK. Impact of cataract and trichiasis on daily activities in a trachoma-endemic area. *Invest Ophthalmol Vis Sci* 2004; **45**(5): 1061-.

35.	Wolle MA, Cassard SD, Gower EW, et al. Impact of Trichiasis surgery on physical functioning in Ethiopian patients: STAR trial. *Am J Ophthalmol* 2011; **151**(5): 850-7.

36.	Rajak SN, Habtamu E, Weiss HA, et al. Surgery Versus Epilation for the Treatment of Minor Trichiasis in Ethiopia: A Randomised Controlled Noninferiority Trial. *PLoS Med* 2011; **8**(12): e1001136.

37.	Woreta TA, Munoz BE, Gower EW, Alemayehu W, West SK. Effect of trichiasis surgery on visual acuity outcomes in Ethiopia. *Archives of ophthalmology (Chicago, Ill : 1960)* 2009; **127**(11): 1505-10.

38.	Grosh ME, Glewwe P, Reconstruction IBf, Development. Designing household survey questionnaires for developing countries: lessons from 15 years of the Living Standards Measurement Study: World Bank Washington, DC; 2000.

39.	Burton MJ, Mabey DC. The global burden of trachoma: a review. *PLoSNeglTropDis* 2009; **3**(10): e460.

40.	Frick KD, Hanson CL, Jacobson GA. Global burden of trachoma and economics of the disease. *AmJ TropMedHyg* 2003; **69**(5 Suppl): 1-10.

41.	West SK, West ES, Alemayehu W, et al. Single-dose azithromycin prevents trichiasis recurrence following surgery: Randomized trial in ethiopia. *Archives of Ophthalmology* 2006; **124**(3): 309-14.

42.	Polack S, Kuper H, Mathenge W, Fletcher A, Foster A. Cataract visual impairment and quality of life in a Kenyan population. *British Journal of Ophthalmology* 2007; **91**(7): 927-32.

43.	Harper A, Power M. Development of the World Health Organization WHOQOLBREF quality of life assessment. *Psychological medicine* 1998; **28**.

44.	United Nations. World Summit for Social Development. Copenhagen, Denmark: United Nations, 1995.

45.	Dawson CR, Jones BR, Tarizzo ML. Guide to Trachoma Control. Geneva: World Health Organization; 1981.

# Appendices

## Appendix 1: Standard Operating Procedure for Taking Informed Consent

1. **Purpose**

To describe the standard operating procedure for taking informed consent; so that each and every participant will be enrolled in the trial with full knowledge and understanding of what the project is about and its benefits and risks.

2. **Introduction**

After the patient is triaged and identified as eligible participant, s/he will be asked to provide consent for participation and has to go through the following informed consent procedure.

3. **Responsible staff**

- Consent and questionnaire field workers
- Field coordinator (TW)

4. **Procedure**
   4.1. ***General***
        4.1.1. Make sure you have pencil/pen/pencil sharpener and eraser and prepare the trial patient registration logbook (Please use the format shown at the end of this SOP)
        4.1.2. Prepare blood pressure (BP) measurement instruments
        4.1.3. Greet participant, introduce yourself, and make the patient at ease.
        4.1.4. Measure the blood pressure of the patient
               4.1.4.1. If BP systolic >170, diastolic >110 explain to the patient that he/she is medically unfit for the surgery and provide him referral slip to seek medical help
               4.1.4.2. If BP systolic <170, diastolic <110 proceed and take informed consent
        4.1.5. Check if patient heard and understood initial lecture. If not understood, explain about trachomatous trichiasis, blindness and the nature of the trial.
        4.1.6. If the patient is not able to read or understand the information provided, witnessed explanation should be done
   4.2. ***Provide the necessary information***
        4.2.1. Explain about the need for consent in the trial.
        4.2.2. Make sure they understand what consent is and why it is needed. Ensure there are no misconceptions about why consent is being taken, e.g. that the form is related to voting or government.
        4.2.3. If patient can read, provide Information sheet to read. If not, then read to the patient.
        4.2.4. Invite the patient to ask questions that need clarification
   4.3. ***Check the patient’s understanding***
        4.3.1. Ask the patient the following questions to confirm that he/she has understood the information
               4.3.1.1. What is the cause of trachoma
               4.3.1.2. What causes the blindness in trachoma
               4.3.1.3. What is the treatment to prevent the blindness from trachoma
               4.3.1.4. What is this study about
               4.3.1.5. What benefits are there to take part in this study
               4.3.1.6. Are there any risks to take part in this study
               4.3.1.7. Why are we requesting you for your consent
   4.4. ***Take consent***
        4.4.1. Ask the patient if he/she agrees to be enrolled in the trial
        4.4.2. If patient is willing to participate in the trial, read the patient the 5 points specified on the consent form one by one and ask if the patient agreed on each point
        4.4.3. If the patient agreed in each point, tick the boxes on the right hand side
        4.4.4. For a patient to be enrolled in the study, he/she has to agree in all 5 points
        4.4.5. Ask them to sign on two copies of the consent form with signature or thumb print
        4.4.6. Provide the patient a unique reference number and write name of the patient and date the consent is provided (signed) on the two consent forms
        4.4.7. If witnessed informed consent is taken, ask the witness to sign in the two consent forms
        4.4.8. Make sure you (the consent field worker) signed on the two consent form and document the date the consent is taken
        4.4.9. Provide one copy of the completed and signed consent form to the patient and keep the second signed form for the study record
        4.4.10. Collect the treatment/trial eye indicator card from the patient, register demographic detail of the patient and the trial and treatment eye in the log book (See #6) as well as in the patient record form
   4.5. ***Proceed to interview***
5. **Procedure for patients declining consent**
   5.1. If patient is eligible for trial but not willing to participate, discuss case with field co-ordinator
        5.1.1. The field coordinator should discuss with the patient and find out the reasons for not providing consent and answers any questions that the patient raises
   5.2. If the patient agrees to participate and consent, follow instructions under #4
   5.3. If patient does not consent after discussion with the field coordinator or if a patient is not competent to consent:
        5.3.1. Patient becomes non-trial patient
        5.3.2. Continue as per local protocol, ensuring that patient is still offered surgery by the project team
6. **Trial patients’ log**

| SN | Date | Study reference number | Name | Age | Sex | Treatment eye (R/L/ B) | Trial eye | Comparison patient (Yes/No) | Date for household interview | Remark |
| --- | --- | --- | --- | --- | --- | --- | --- | --- | --- | --- |
| 1 |  |  |  |  |  |  |  |  |  |  |
| 2 |  |  |  |  |  |  |  |  |  |  |
| 3 |  |  |  |  |  |  |  |  |  |  |
| 4 |  |  |  |  |  |  |  |  |  |  |

## Appendix 2: Standard Operating Procedure for Clinical Examination

**1. Purpose**

To describe the standard operating procedure to conducting baseline ocular examination prior to surgical treatment in trail patients

1. **Introduction**

Immediately after baseline examination, TT patients (cases) will be randomised (1:1) to receive surgery using either PLTR procedure or BLTR procedure. This requires generating a randomization sequence for each patient

2. **Objectives**

The objective of this SOP is to describe the manners and steps to be followed during examination and define trachoma examination grading

3. **Responsible staffs**

- Trial manager
- Examination assistant

4. **Examination procedure**
   4.1. ***General***
        a. Make sure you have the necessary examination and documentation materials

- Examination magnifying loop
- Examination light/torch
- Examination glove
- Alcohol gel/swab to clean hand between examinations
  b. Explain to the patient that you are going to examine his eyes for sign of trachoma and this does not take too long
  c. Tell the patient that you will use torch light to do the examination
  d. Explain that you will apply local anaesthetics drop in his eye to comfort him/her during the examination
  e. Check that the trial and treatment eyes are written at the beginning of the examination record form
  f. Apply one drop of local anaesthetics on the lower lid fornix of both eyes
  g. Start and finish examining the right eye and then the left eye
  4.2. ***Assess trichiasis (Q9.3-9.9)***
       a. Assess eyelash position with eye in primary position (looking straight ahead) and document numbers in the space provided
          a. Number of lashes whose point touches globe medial to cornea
          b. Number of lashes whose point touches globe lateral to cornea
          c. Number of lashes whose point touches cornea
       b. Determine grade of trichiasis (Q9.6)
          a. Use the following grading definition

| **Trichiasis Grade** | **Definition** |
| --- | --- |
| T 0 | No trichiasis |
| T 1 | Lashes deviated towards the eye, but not touching the globe |
| T 2 | Lashes touching the globe but not rubbing the cornea. |
| T 3 | Lashes constantly rubbing the cornea. |

c. Count and document the different types of lashes (Q9.7 & 9.8)
   a. Count number of metaplastic lashes right and left eye (*i.e. trichiasis not secondary to entropion*)
   b. Count number of misdirected lashes right and left eye
d. Count and document lower lid trichiatic lashes (Q9.9)
4.3. ***Assess entropion (Q9.10)***
     a. Assess orientation of the lid margin of the eye in the primary position
     b. If necessary, gently raise any excess folds of upper lid skin, *without*, disturbing the position of the upper lid.
     c. If there is a mixed picture, classify as the worse grade

| **Degree of severity** | **Area of entropion** | |
| --- | --- | --- |
|  | **<50% of lid margin** | **>50% of lid margin** |
| None | E0 (none) | |
| Without corneal-lash base contact | E1 (mild) | E2 (moderate) |
| With corneal-lash base contact | E3 (severe) | E4 (total) |

d. Use the following grading
e. The grading are defined as follows	‘
   a. None = normal’ lid margin (see glossary) visible
   b. Without corneal-lash base contact = Definite inwards rotation of the lid	margin, without any lash bases touching the cornea
   c. With corneal-lash base contact = Inward rotation of lid margin, with	some or all of the lash bases touch the cornea.
4.4. ***Assess epilation (Q 9.11 & 9.12)***
     a. Assess and document if there is clinical evidence of epilation
        a. Clinical evidence of epilation is defined as presence of broken or short eyelashes or presence of part of the eyelid margin with no lashes
     b. Asses and document the amount of epilation
        a. Examine if evidence of epilation is present in <1/3^rd^ or 1/3^rd^-2/3^rd^ or >2/3^rd^ of the lash margin
     c. Ask the photographer to take trichiasis photo
4.5. ***Assess the eye for presence and type ocular discharge (Q9.13) and document***
     a. Serous = Watery discharge, Purulent = yellowish relatively thick discharge, Foamy = Foam like discharge
4.6. ***Examine corneal scarring (Q 9.14)***
     a. Ask the patient to look straight, and make all grading while the patient is looking in a straight gaze
     b. Shine the torch light on to the cornea without creating glare on the cornea surface
     c. If there are more than one corneal scars, grade as for worst/most central scar
     d. See card below for diagrammatic representations of corneal scar grade

e. Definitions of grading

C1	Opacity not entering central 4mm

C2a	Opacity within central 4mm but not entering within the central 1mm of	the cornea. The pupil	margin is visible through the opacity.

C2b	Opacity within central 4mm but not entering within the central 1mm of	the cornea. The pupil margin is not visible through the opacity.

C2c	Opacity within central 4mm and entering the central 1mm of the	cornea. The pupil margin is visible through the opacity.

C2d	Opacity within central 4mm and entering within the central 1mm of the	cornea. The pupil margin is not visible through the opacity.

C3	Opacity large enough and dense enough to make whole pupil margin	invisible.

C4	Phthisis

f. Ask the photograph filed worker to take corneal scare photo
4.7. ***Assess and document for the presence of lagophthalmos***
     a. Ask the patient to close his/her eyes gently, like he/she is sleeping
     b. Look for the closure of the lids
     c. Lagophthalmos is defined as “the inability to close the eyelids completely”.
4.8. **Assess the upper tarsal conjunctiva for the following Signs**
     a. Ask the patient to look down
     b. Gently pull and evert the upper eyelid, at the same time by applying a slight pressure at approximately the centre of the eyelid externally
     c. Assess the presence of conjunctivalisation of the lid margin (Q 9.18)
        a. Use the following grading and definition

CM 0	No conjunctivalisation of the lid margin

CM 1	The muco-cutaneous junction is located anterior to its normal position,	but the whole line is still posterior to the line of Meibomian gland	orifices.

CM 2	The muco-cutaneous junction is located anterior to the line of the	Meibomian gland orifices for less than 50% of the lid.

CM 3	The muco-cutaneous junction is located anterior to the line of the	Meibomian gland orifices for greater than 50% of the lid.

d. Examine upper lid papillary hypertrophy (Q 9.19)
   a. Determine the grade of severity of papillary hypertrophy using the following definitions

P 0	Absent: normal appearance

P1	Minimal: individual vascular tufts (papillae) prominent, but deep	subconjunctival vessels on the tarsus are not obscured.

P2	Moderate: more prominent papillae and normal vessels appear hazy,	even when seen by the naked eye.

P3	Pronounced: conjunctiva thickened and opaque, normal	vessels on the tarsus are hidden over more than half of the surface.

b. Determine whether papillary reaction is over less than or greater than 50% of the tarsal area using the following grading

|  | <50% of tarsal area inflamed | >50% of tarsal area inflamed |
| --- | --- | --- |
| None | 0 | 0 |
| Mild | 1 | 2 |
| Moderate | 3 | 4 |
| Severe | 5 | 6 |

c. To convert to traditional papillary grading system use the following table

| Degree of inflammation /’redness’ | <50% of tarsal area inflamed | >50% of tarsal area inflamed |
| --- | --- | --- |
| None | P0 | P0 |
| Mild | P1 | P1 |
| Moderate | P2 | P2 |
| Severe | P2 | P3 |

e. Assess conjunctival scarring (Q 9.20)
   a. Use the following grading and definition if there is no previous lid surgery (Q 9.20)

C0	No scarring on the conjunctiva

C1	Mild: fine scattered scars on the upper tarsal conjunctiva, or scars on	the other parts of the conjunctiva

C2	Moderate: more severe scarring but without shortening or distortion of	the upper tarsus.

C3	Severe: scarring with distortion of the upper tarsus.

C6	Not applicable

b. Use the following grading if there is previous lid surgery (Q 9.21)

SCO	No scarring on the conjunctiva

SC1	Surgical line only.

SC2	Surgical line and occasional scattered scars

SC3	Surgical scar with widespread trachomatous scarring but no distortion

SC4	Surgical scar with distortion immediately around the incision line.

SC5	Surgical scar with additional distortion secondary to widespread	trachomatous scarring.

SC6	Not applicable

c. Ask the photographer to take tarsal conjunctiva photo
4.9. ***Document if you noticed other conditions during the examination (Q 9.23)***

5. **Complete ocular examination**
   5.1. Check the examination data record sheet for completeness before the patient leaves the room
   5.2. Ask the photographer to check that he has all the photographs with the quality needed
   5.3. Thank and tell the patient that the examination is completed
   5.4. Tell the examination assistant to transfer the patient to the surgery waiting area and the data record form to the randomization administrator

## Appendix 3: BLTR Surgical Procedure

1. **Introduction**

The standard surgical procedure for the Bilamellar Tarsal Rotation surgery is described in detail in the WHO/PBL “Trichiasis Surgery for Trachoma: The Bilamellar Tarsal Rotation Procedure” Manual. The trial surgeons will receive a practical training on BLTR procedure by ophthalmologists. The exact procedures to be used for the BLTR and are those described in detail in the above WHO trichiasis surgery manual. After completing the training and standardization process, the surgeons will return to their area and will perform at least 50 surgeries using each procedure before they are reassessed by three experienced ophthalmologists as per the WHO manual for “final assessment of trichiasis surgeons”.

2. **Objectives**

This summary of the BLTR procedure has the following objectives:

- Will be used by the trial surgeons a s a quick reference when needed
- Will be used as a checklist to follow that the trial surgeons are operating as per standards

3. **Responsible staff**
   - Trial trichiasis surgeons
4. **Summary of the BLTR procedure**
   4.1. ***General***
        a. Refer to the manual for detailed aseptic technique.
        b. Clean the face particularly the lids and the eyebrow area using cotton or gauze soaked in 10% Povidone Iodine or other skin preparation.
   4.2. ***Anaesthetic injection:***
        a. Using a new sterile needle and syringe, draw 3-5 ml of 2% lidocaine with adrenalin.
        b. Ask the patient to look down and Insert the needle in the plane of the upper eye lid temporal from the lateral canthus and 3 mm above the lid margin.
        c. Maximum care should be taken to avoid poking the needle out through the eyelid or piercing the eyeball. Inject the anaesthetic ahead of the sliding needle, or slide the needle and withdraw, injecting the anaesthetic continuously.
        d. Massage the lignocaine in to the eyelid with for one minute with swab and gentle figure pressure. Wait three minutes until the lignocaine has take effect.
   4.3. ***Fixing the eye Lid:***
        a. Place a haemostat at the upper medial end of the upper lid, just lateral to the lachrymal punctum, and close with moderate pressure.
           a. It should extend 5 mm in from the lid margin.
        b. Place another haemostat at the lateral end of the upper lid, angled outwards,, also extending 5 mm in from the lid margin.
        c. Confirm that the lid can be everted without difficulty.
        d. The haemostat should not be closed on the lid for more than 15 minutes: interrupts blood flow of the lid.
        e. Alternatively, a TT/Waddell clamp can be used to replace the 2 hemostats used to fix the eyelid. The lid clamp provides an integrated eyelid plate which prevents the risk of piercing the eyeball and allows a single full thickness incision.
        f. If using the TT or Waddell clamp, haemostats are not used. The TT clamp

is placed so that the eyelid margin lines up with the groove on the plate and then secured. The Waddell clamp is placed so that the eyelid margin is up against the vertical piece on the clamp and then secured. The plate between the eyelid and the eye allows a full thickness incision to be made with either clamp.

4.4. ***Incising the eyelid***
     a. Incising the skin and the muscle
        a. Hold the haemostat downwards so that the eyelid does not move.
        b. Incise the skin and muscle parallel to the lid margin and 3mm above it, the entire distance between the haemostats.
        c. The blade is held at right angles to the skin, and enters to a depth just superficial to the tarsal plate.
     b. Incise the conjunctiva and the tarsal plate
        a. Evert the eyelid
        b. Incise the lid margin and 3mm above it. The entire distance between the haemostats.
     c. If using the TT or Waddell clamp, the incision is made “full thickness” or through all layers down to the metal plate from one side of the clamp to the other
4.5. ***Untie the incisions***
     a. Elevate the lid with the haemostats.
     b. Insert the points of the closed scissors into the incision in the conjunctiva-tarsal plate, through remaining intact muscle, and out through the skin-muscle incision.
     c. Open the scissors while still held across the lid: the blunt aspects of the blades will spread apart intact muscle.
     d. Remove the haemostats. The lid may bleed profusely. Stop the bleeding by firm pressure with a sterile swab for one minute.
4.6. ***Complete the incision medially and laterally***
     a. Open the incision by grasping and elevating the skin of the lid margin, near where you intend to cut, with toothed forceps.
     b. Using the scissors completely divide the medial and lateral edges of the tarsal plate (the portion formerly held in the haemostats), still cutting parallel to the lid margin.
     c. Do not cut much beyond the edge of the tarsal plate medially as the marginal artery may be cut and bleed.
     d. The eyelid should now be divided through its entire thickness, 3 mm from and parallel to the lid margin, remaining connected at both ends.
     e. The 3mm lid margin portion is referred to as the distal fragment, the remaining portion as the proximal fragment.
4.7. ***Suturing the eyelid***
     a. Placing suture in the proximal fragment:
        a. The sutures need needles at both sides.
        b. Mount the needle to point towards you.
        c. Grasp the cut edge of the tarsal plate with toothed forceps.
        d. Observe the pink conjunctiva on the inner surface of the eyelid.
        e. Pass the first needle and its associated suture through 1mm bite of tarsal conjunctiva and ¼ of the thickness of the tarsal plate, near the middle of the eyelid.
        f. Note that the needle emerges from the cut edge of the tarsal plate.
        g. Pass the second needle, at the other end of the same suture, through the conjunctiva and the tarsal plate in the same way, so that the suture is symmetrically placed at the centre of the eyelid.
        h. Place a haemostat on the two strands of suture.
        i. Pass double –armed suture in an identical manner on either side of the first.
        j. They must reach the medial and lateral ends of the incision. Otherwise trichiasis will return at either side.
        k. If using the Waddell clamp, place the stitches first, then remove the clamp and tighten the sutures
     b. Placing sutures in the distal fragment
        a. Mount the needle to point away from you.
        b. Grasp the skin of the distal fragment of eyelid.
        c. Pass the needle through the muscle layer on the front surface of the tarsal plate to emerge through the skin about 1mm above the eyelashes.
        d. The entry point should correspond with the site of the suture in the proximal eyelid fragment.
        e. Repeat this with a second needle on the same suture.
        f. Clip the two ends of the suture.
        g. Repeat this with the two other sutures on the medial and lateral sides.
     c. Tying the sutures:
        a. Tie the central suture with three singe knots.
        b. Then tie the other two sutures in the same way.
        c. They should be tied firmly enough to produce a slight overcorrection.
        d. Cut the sutures 3 mm above the knot. This is long enough to permit ready removal, without being so long as to irritate the eye.
        e. The final result should show and eyelid with a slight overcorrection.
        f. The eyelashes should point well way from the eye all along the edge of the eyelid.
4.8. ***Applying antibiotic and dressing***
     a. Apply tetracycline eye ointment into conjunctival sac and onto the wound.
     b. Pad the eye
        a. A bandage may also be used.
     c. Give two 500 mg tablets of Paracetamol for pain.
4.9. ***Post OP advise***
     a. Advise the patient to stay quietly at home for one or two days
     b. To open the eye pad after 12 hours and clean the face
     c. Demonstrate to the patient and/or his relative how to apply the antibiotic ointment particularly the amount and the part of the eye (conjuctival sac) it should be applied; and instruct clearly on the dosage frequency and make sure the patient and the relative understood these.
     d. Provide appointment card for follow-up and explain when and where to come
     e. Thank and discharge

## Appendix 4: PLTR Surgical Procedure

1. **Introduction**

The standard surgical procedure for the Bilamellar Tarsal Rotation surgery is summarised in the WHO manual for “Final Assessment of Trichiasis Surgeons”. The PLTR surgery will be performed by certified, experienced trichiasis surgeons (IECWs) working in the trachoma control programme in Amhara Region using the PLTR procedure. The surgeons will receive practical standardization training on PLTR procedure by ophthalmologists and will be assessed by three experienced ophthalmologists as per the WHO manual for “final assessment of trichiasis surgeons”.

2. **Objectives**

This summary of the PLTR procedure has the following objectives:

- Will be used by the trial surgeons a s a quick reference when needed
- Will be used as a checklist to follow that the trial surgeons are operating as per standards

3. **Responsible staff**
   - Trial trichiasis surgeons
4. **Summary of the PLTR procedure**
   4.1. ***General***
        a. Refer to the WHO yellow manual for detailed aseptic technique.
        b. Clean the face particularly the lids and the eyebrow area using cotton or gauze soaked in 10% Povidone Iodine or other skin preparation.
   4.2. ***Anaesthetic injection***
        a. Using a new sterile needle and syringe, draw 3-5 ml of 2% lidocaine with adrenalin.
        b. Ask the patient to look down and Insert the needle in the plane of the upper eye lid temporal from the lateral canthus and 3 mm above the lid margin.
        c. Maximum care should be taken to avoid poking the needle out through the eyelid or piercing the eyeball. Inject the anaesthetic ahead of the sliding needle, or slide the needle and withdraw, injecting the anaesthetic continuously.
        d. Massage the lignocaine in to the eyelid with for one minute with swab and gentle figure pressure. Wait three minutes until the lignocaine has take effect.
   4.3. ***Application of traction suture***
        a. The lid should not be everted at the start.
        b. Approximately 3 mm from the upper lid margin, insert the needle with suture through the skin and orbicularis, starting either laterally or medially.
        c. Take two large bites, about 5 mm in length, with a similar space in between. There should be a loop in the middle.
   4.4. ***Everting the lid and keeping it in position***
        a. Sling the suture loop into a Trabut entropion plate or Wilde’s entropion forceps, pulling the two suture ends and at the same time everting the lid.
        b. Use the forceps as a fulcrum as the lid is everted.
        c. The lid is kept in the everted position by clamping the suture to the drape using the haemostat forceps.
   4.5. ***Incision of tarso-conjunctiva***
        a. Using the blade, scratch an incision along the Arlt’s line (3-4 mm from the margin) and deepen the cut until the orbicularis muscle is reached (through the tarsal plate).
        b. Extend the incision over the whole length of the tarsal plate, ending just before the lachrymal punctum medially and laterally at the canthus.
        c. Place a tissue forceps on the proximal tarsal conjunctiva to hold the incised tarso-conjunctiva, and complete the incision with scissors.
   4.6. ***Blunt dissection of the tarso-conjunctiva***
        a. Pick up the tarso-conjunctiva with tissue forceps.
        b. Insert closed scissors and spread them, dissecting the tarso-conjunctiva from the overlying orbicularis muscle.
        c. The dissection should extend for approximately 8 mm.
   4.7. ***Suturing to achieve eversion of the distal fragment of the lid margin***
        a. Use mattress sutures, taking 1-mm bites of tarsal conjunctiva and half the thickness of the tarsal plate, passing the needle under the distal tarsal conjunctiva and emerging through the skin about 3 mm above the lid margin.
        b. A minimum of three or four evenly spaced mattress sutures are to be applied.
        c. To finish, all the sutures are pulled up together so as to bury the proximal fragment edge of the tarso-conjunctiva under the distal fragment.
        d. Starting in the middle, tie the sutures snugly with three single knots, and cut 3 mm above the knot.
   4.8. ***Remove the traction suture and the Wilde’s entropion forceps or Trabut plate***.
   4.9. ***Applying antibiotic and dressing***
        a. Apply tetracycline eye ointment into conjunctival sac and onto the wound.
        b. Pad the eye
           a. A bandage may also be used.
        c. Give two 500 mg tablets of Paracetamol for pain.
   4.10. ***Post OP advise***
         a. Advise the patient to stay quietly at home for one or two days
         b. To open the eye pad after 12 hours and clean the face
         c. Demonstrate to the patient and/or his relative how to apply the antibiotic ointment particularly the amount and the part of the eye (conjuctival sac) it should be applied; and instruct clearly on the dosage frequency and make sure the patient and the relative understood these.
         d. Provide appointment card for follow-up and explain when and where to come
         e. Thank and discharge

## Appedix 5: Adverse Event Reporting Protocol

|  | | | | | | | | | | AE observed | | | | | | | | | | | | |  | | | | | | | | |
| --- | --- | --- | --- | --- | --- | --- | --- | --- | --- | --- | --- | --- | --- | --- | --- | --- | --- | --- | --- | --- | --- | --- | --- | --- | --- | --- | --- | --- | --- | --- | --- |
|  | | | | | | | | | | | | | | | | | | | | | | | | | | | | | | | |
|  | | | | | | | | | | Is it serious? | | | | | | | | | | | | |  | | | | | | | | |
|  | | | | | | | | | | | | | | | | | | | | | | | | | | | | | | | |
|  | | | | | | | Yes | | | | | | | | |  | | | | | No | | | | | |  | | | | |
|  | | | | | | | | | | | | | | | | | | | | | | | | | | | | | | | |
|  | | | Is it expected, ie included in the IMP’s Investigator Brochure or Summary of Product Characteristics? | | | | | | | | | | | |  | | | | | | | | | | | AE. It does not require expedited reporting. Describe on CRFs | | | | |  |
|  | | | | | | | | | | | | | | | | | | | | | | | | | | | | | | | |
|  | | Yes | | |  | | | | | | | | No | | | |  | | | | | | | | | | | | | | |
|  | | | | | | | | | | | | | | | | | | | | | | | | | | | | | | | |
| SAE. Assess for severity, complete SAE form and report to PI within 24 hours To go on annual safety report | | | | | | | |  | | | | | | Is it reasonably, causally related to the study intervention or trial medication? | | | | | | | | | | | | |  | | | | |
|  | | | | | | | | | | | | | | | | | | | | | | | | | | | | | | | |
|  | | | | | | | | | | | | Yes | | | |  | | | | | | | | | No | | | |  | | |
|  | | | | | | | | | | | | | | | | | | | | | | | | | | | | | | | |
| AE=Adverse Event  SAE=Serious Adverse Event  SUSAR= Suspected Unexpected SAE | | | | | | SUSAE. Report to CI within 24 hours. Requires expedited reporting to NRERC, Ethiopia; FMHACA, DSMB, LSHTM/ESTC Health Research Department | | | | | | | | | | | | | |  | | | | SAE. Assess for severity, complete SAE form and report to PI within 24 hours. To go on annual safety report | | | | | | |  |
|  | | | | | | | | | | | | | | | | | | | | | | | | | | | | | | | |
|  | | | | | | | | | Life-threatening or fatal? | | | | | | | | |  | | | | | | | | | |  | | | |
|  | | | | | | | | | | | | | | | | | | | | | | | | | | | | | | | |
|  | | | | Yes | | | | | | |  | | | | | | No | | | | |  | | | | | | | | | |
|  | | | | | | | | | | | | | | | | | | | | | | | | | | | | | | | |
|  | Report to NRERC, Ethiopia; FMHACA, DSMB, LSHTM/ESTC Health Research Department  within 7 days | | | | | | | |  | | | | | | | | | | Report to NRERC, Ethiopia; FMHACA, DSMB, LSHTM/ESTC Health Research Department  within 15 days | | | | | | | | | | |  | |

## Appendix 6: SAE reporting Format

London School of Hygiene & Tropical Medicine	E4

Keppel Street, London WC1E 7HT	Oct 2011

Observational/Interventions Research Ethics Committee

Serious Adverse Events Report (Expedited Report)

*Please ensure you download and complete the latest version of this form from the intranet:* [*http://intra.lshtm.ac.uk/management/committees/ethics/*](http://intra.lshtm.ac.uk/management/committees/ethics/)

All serious adverse events concerning a study approved by the LSHTM Observational/Interventions Research Ethics Committee should be reported to the Committee and other relevant bodies. A serious adverse event may include Suspected Unexpected Serious Adverse Reactions (SUSARs) or violations/serious breaches of the protocol.

*This form should be completed and emailed along with all relevant attachments to* [*ethics@lshtm.ac.uk*](mailto:ethics@lshtm.ac.uk)*.*

| For use of Ethics Committee only | Report No. | Date received |
| --- | --- | --- |

| Title of Project | |
| --- | --- |
| LSHTM Ethics Committee  Reference number |  |
| Date approved |  |
| Name of Principal Investigator (PI)  at LSHTM |  |
| Appointment Held (or Research student) |  |
| Department/Faculty |  |
| Date of event: |  |
| Date of first awareness of event: |  |
| Date form completed: |  |

Describe the serious adverse event

Describe impact on the study, including where temporary halt to activities took place

Describe management and follow-up of the serious adverse event:

Describe steps to avoid recurrence (if possible):
