# Supplementary material for: Posterior lamellar versus bilamellar tarsal rotation surgery for trachomatous trichiasis: Long-term outcomes from a randomised controlled trial
Source: eClinicalMedicine. 2019 Nov 1;17:100202. doi: 10.1016/j.eclinm.2019.10.015 (PMC6933225; doi:10.1016/j.eclinm.2019.10.015)
Supplement: Supplementary Data S1 — Supplementary Raw Research Data. This is open data under the CC BY license http://creativecommons.org/licenses/by/4.0/ [file mmc1.zip › S2589537019302019.zip!/coi_disclosure BG.pdf]

Please wait...

If this message is not eventually replaced by the proper contents of the document, your PDF viewer may not be able to display this type of document.

You can upgrade to the latest version of Adobe Reader for Windows®, Mac, or Linux® by visiting http://www.adobe.com/go/reader_download.

For more assistance with Adobe Reader visit http://www.adobe.com/go/acrreader.

Windows is either a registered trademark or a trademark of Microsoft Corporation in the United States and/or other countries. Mac is a trademark of Apple Inc., registered in the United States and other countries. Linux is the registered trademark of Linus Torvalds in the U.S. and other countries.
