# Supplementary material for: Posterior lamellar versus bilamellar tarsal rotation surgery for trachomatous trichiasis: Long-term outcomes from a randomised controlled trial
Source: eClinicalMedicine. 2019 Nov 1;17:100202. doi: 10.1016/j.eclinm.2019.10.015 (PMC6933225; doi:10.1016/j.eclinm.2019.10.015)
Supplement: Supplementary Data S1 — Supplementary Raw Research Data. This is open data under the CC BY license http://creativecommons.org/licenses/by/4.0/ [file mmc1.zip › S2589537019302019.zip!/ecm-author-signature_TW & BG.pdf]

EClinicalMedicine
Published by THE LANCET

Author statements

Please insert the relevant text under the subheadings below. A completed form must be signed by all authors. Please note that we will accept hand-signed and electronic (typewritten) signatures. Please complete multiple forms if necessary, and upload the signed copy with your submission.

Manuscript title: Posterior lamellar versus bilamellar tarsal rotation surgery for trachomatous trichiasis: long-term outcomes from a randomised controlled trial

Corresponding author: Dr Esmael Habtamu

Article type: Clinical Trial

Does your manuscript have a reference number? No ○ Yes ○ If yes, enter number here: ECLINM-D-19-00219

Does your manuscript have a handling editor? No ○ Yes ○ If yes, enter name here: Arianna Colosio

Authors' contributions

Please insert here the contribution each author made to the manuscript—eg, literature search, figures, study design, data collection, data analysis, data interpretation, writing etc. If all authors contributed equally, please state this. The information provided here must match the contributors' statement in the manuscript.

Literature search: EH MJB
Study conception and design: EH ZT EKC DM MJB
Data collection: EH TW ZT BA BG
Statistical analysis: EH DM
Data interpretation: EH DM MJB
Drafting the manuscript: EH
Critical revision of the manuscript for important intellectual content: All authors
Obtained funding: EH MJB
Administrative, technical or material support: TW ZT BA BG AG KC
Study supervision: ZT BG MJB

EClinicalMedicine
Published by THE LANCET

Role of the funding source

Please disclose any funding sources and their role, if any, in the writing of the manuscript or the decision to submit it for publication. Examples of involvement include: data collection, analysis, or interpretation; trial design; patient recruitment; or any aspect pertinent to the study. Please also comment whether you have been paid to write this article by a pharmaceutical company or other agency. If you are the corresponding author, please indicate if you had full access to all the data in the study and had final responsibility for the decision to submit for publication. The information provided here must match the role of the funding source statement in the manuscript.

The fourth-year follow-up received financial support from the Coalition for Operational Research on Neglected Tropical Diseases (COR-NTD), at the Task Force for Global Health. EH is supported by the Queen Elizabeth Diamond Jubilee Trust. MJB and the initial trial was funded by The Wellcome Trust.

The funders had no role in study design, data collection and analysis.

Conflicts of interest

Please complete the ICMJE conflict of interest form, which is available at http://www.thelancet.com/for-authors/forms#icmje-coi. Please ensure that a conflict of interest statement is included at the end of the manuscript, which matches what is declared on the ICMJE conflict of interest form.

Patient consent (if applicable) - completion of this section is mandatory for Case Reports, Clinical Pictures, and Adverse Drug Reactions.
Please sign below to confirm that all necessary consents required by applicable law from any relevant patient, research participant, and/or other individual whose information is included in the article have been obtained in writing. <u>**The signed consent form(s) should be retained by the corresponding author and NOT sent to *EClinicalMedicine*.**</u>

I agree with: the plan to submit to *EClinicalMedicine;* the contents of the manuscript; to being listed as an author; and to the conflicts of interest statement as summarised. I have had access to all the data in the study (for original research articles) and accept responsibility for its validity.

Title and name:	Highest degree:	Signature:	Date:
Dr Esmael Habtamu	PhD	Esmael H	23/08/19
Mr Tariku Wondie	BA	Tariku	03/09/19
Dr Zerihun Tadesse	MD		
Sr Bezawit Atinafu	BSc		
Mr Bizuayehy Gashaw	MHA	Bizuayehu	03/09/19
Dr Abebaw Gebeyehu	PhD		
Ms E.Kelly Callahan	MPH		
Dr David Macleod	PhD		
Prof Matthew J Burton	PhD		

Corresponding author declaration

I Dr Esmael Habtamu, the corresponding author of this manuscript, certify that the contributors' and conflicts of interest statements included in this paper are correct and have been approved by all co-authors.
