# Supplementary material for: Posterior lamellar versus bilamellar tarsal rotation surgery for trachomatous trichiasis: Long-term outcomes from a randomised controlled trial
Source: eClinicalMedicine. 2019 Nov 1;17:100202. doi: 10.1016/j.eclinm.2019.10.015 (PMC6933225; doi:10.1016/j.eclinm.2019.10.015)
Supplement: Supplementary Data S1 — Supplementary Raw Research Data. This is open data under the CC BY license http://creativecommons.org/licenses/by/4.0/ [file mmc1.zip › S2589537019302019.zip!/Research in context_v2.docx]

**Research in context:**

**Evidence before this study**

Our study group conducted and published a systematic review of the management of trachomatous trichiasis searching CENTRAL, Ovid MEDLINE, EMBASE, ISRCTN registry, ClinicalTrials.gov, WHO ICTRP. The only randomised trial which compared variants of the Bilamellar Tarsal Rotation (BLTR) and Posterior Lamellar Tarsal Rotation (PLTR) surgical procedures performed by ophthalmologists in a teaching hospital in Ethiopia was conducted in 2002, which found no evidence of a difference in outcome after three months. We conducted a randomised, controlled, single masked clinical between February and May 2015 to determine the relative effectiveness of the PLTR and BLTR Ethiopia in larger sample (1000 patients) in a programmatic setting in Ethiopia. The 12-month results showed that the PLTR was superior to BLTR giving a substantially lower trichiasis recurrence rate by one year and fewer intra and immediate post-operative complications. There is no data on the long-term outcome of these two surgical procedures from a head to head comparison trial.

**Added value of this study**

The one-year results of our trial led to a shift in international guidance from BLTR being the treatment of choice to treat trachomatous trichiasis to a preference for new trainees to be taught PLTR. However, there was much international interest to see the long-term outcome of the PLTR and BLTR. Our trial participants were examined four years after randomised intervention to assess the long-term outcome of these two procedures and to ascertain whether the superiority of PLTR would be sustained beyond one year. The results showed that the PLTR surgical procedure had still superior long-term outcomes to the BLTR with significantly lower risk of recurrent trichiasis four years after surgery.

**Implications of all the available evidence**

The available evidences support the current WHO guideline that the PLTR should be the procedure of choice for training new surgeons in the programmatic management of TT.
