# Supplementary material for: Posterior lamellar versus bilamellar tarsal rotation surgery for trachomatous trichiasis: Long-term outcomes from a randomised controlled trial
Source: eClinicalMedicine. 2019 Nov 1;17:100202. doi: 10.1016/j.eclinm.2019.10.015 (PMC6933225; doi:10.1016/j.eclinm.2019.10.015)
Supplement: Supplementary Data S1 — Supplementary Raw Research Data. This is open data under the CC BY license http://creativecommons.org/licenses/by/4.0/ [file mmc1.zip › S2589537019302019.zip!/1. RCT BLTR v PLTR - Detailed Study Protocol - Amended 12AUG2017_II.docx]

**Posterior lamellar versus bilamellar tarsal rotation surgery for trachomatous trichiasis: long-term outcomes from a randomised controlled trial**

**Authors**

Esmael Habtamu PhD^1,2^, Tariku Wondie BA^2^, Zerihun Tadesse MD^2^, Bezawit Atinafu BSC^2^, Bizuayehu Gashaw MHA^3^, Abebaw Gebeyehu MD^3^, E. Kelly Callahan MPH^4^, David Macleod PhD^5^, and Matthew J. Burton PhD^1^

**Affiliations**

^1^Clinical Research Department, International Centre for Eye Health, London School of Hygiene & Tropical Medicine, London, UK

^2^ The Carter Center, Addis Ababa, Ethiopia

^3^ Amhara Regional Health Bureau, Bahirdar, Ethiopia

^4^ The Carter Center, Atlanta, USA

^5^ Department of Infectious Disease Epidemiology, London School of Hygiene & Tropical Medicine, London, UK

**Corresponding Author:**

Dr Esmael Habtamu

Clinical Research Department, International Centre for Eye Health

Faculty of Infectious and Tropical Diseases

London School of Hygiene and Tropical Medicine

Keppel Street

London WC1E 7HT

United Kingdom

Email: [esmael.ali@lshtm.ac.uk](mailto:esmael.ali@lshtm.ac.uk)

Telephone: +44 (0)20 7927 2329

Short title: Long-term outcomes of PLTR v BLTR surgical procedures

Keywords: Trachoma, Trichiasis, Ethiopia, Surgery, Postoperative, Recurrence

**Summary**

***Background:*** We re-examined the participants of a clinical trial four years after enrolment to identify which of the two most commonly used eyelid surgery procedures to treat the blinding stage of trachoma (trachomatous trichiasis, TT), the posterior Lamellar Tarsal Rotation (PLTR) and Billamelar Tarsal Rotation (BLTR), gives better results in the long-term.

***Methods:*** A randomised, controlled, single masked clinical trial was done in Ethiopia. At baseline, adults (aged >18 years with upper lid unoperated TT were recruited from a community-based screening. Participants were randomly assigned (1:1), to either BLTR or PLTR surgery, stratified by surgeon. At 4 years an independent assessor masked to allocation examined the trial participants’ eyes using the same procedures as for the baseline and earlier follow-ups. The primary outcome was the proportion of individuals who had recurrence (postoperative TT, PTT) at the 4-year examination, or a history of repeat surgery in the 4-year period. The intervention effect was estimated by logistic regression, controlled for surgeon as a fixed effect in the model. The trial is registered with the Pan African Clinical Trials Registry (number PACTR201401000743135).

***Findings:*** 1000 participants with TT were enrolled, randomly assigned, and treated (501 in the BLTR group and 499 in the PLTR group) between Feb 13, 2014, and May 31, 2014. At year 4, 943 (94.3%) participants were re-examined (471, PLTR; 472, BLTR) and included in the primary outcome analysis. PTT had developed in 169/943 (17·9%) study eyes, among which 129 (76·3%) had minor trichiasis (≤5 lashes touching the eye). PTT was significantly more frequent at 4-year in the BLTR arm (105/472 [22·2%]) than the PLTR arm (64/471 [13·6%]), adjusted OR 1·82 (95% CI, 1·29–2·56); p=0·0006, with 8·6% (95%CI 3·8–13·5) risk difference.

***Interpretation:*** The PLTR surgical procedure had superior long-term outcomes to the BLTR with significantly lower risk of PTT supporting the current WHO guideline that the PLTR should be the procedure of choice for training new surgeons in the programmatic management of TT.

***Funding:*** Coalition for Operational Research on Neglected Tropical Diseases (COR-NTD), at the Task Force for Global Health. The initial trial was funded by The Wellcome Trust (Grant Number 098481/Z/12/Z).

**Introduction**

Trachomatous Trichiasis (TT), the blinding stage of trachoma, is mainly treated with corrective eyelid surgery.^1,2^ Many surgical procedures have been tried for the management of TT.^3^ However, the two most commonly used surgical procedures are the Posterior Lamellar Tarsal Rotation (PLTR) and the Bilamellar Tarsal Rotation (BLTR) surgeries.^2^ The type of surgical procedure is thought to be one of the major determinants of outcome of TT surgery.^4-8^ Poor surgical outcomes pose a major challenge for surgical programmes worldwide. Trichiasis typically recurs in around 20% of patients within a year, and about 10% develop eyelid contour abnormality (ECA).^9-12^ Empirical data indicate that poor surgical outcomes deter patients from accepting trichiasis surgery,^13^ possibly contributing to the recent decline in surgical uptake in some trachoma control programmes.^14^

Four years ago, we conducted a randomised, controlled, single masked clinical trial to compare the relative effectiveness of the PLTR and BLTR procedures. One year after surgery we found that the cumulative rate of recurrent trichiasis (here after postoperative trachomatous trichiasis, PTT) was more frequent in the BLTR group than in the PLTR group with a 9.5% risk difference.^9^ Following this, international guidance on the surgical treatment of choice was updated, shifting away from BLTR being the treatment of choice to a preference for new trainees to be taught PLTR.^15^

There are about 3 million un-operated cases of TT globally,^16^ requiring surgery using the safest and most successful procedure. However, there are currently no long-term data directly comparing these two surgical procedures. Some studies have reported that the rate of PTT may increase from about 20% at 1-year to as much as 60% at 3 years after surgery.^4,5,11,12,17-19^

In this long-term follow-up of a randomised controlled surgical trial, we followed and examined trial participants four years after enrolment to investigate the long-term outcomes of BLTR and PLTR surgery, and to ascertain whether the superiority of the PLTR outcome was sustained beyond one year.

**Methods**

**Study Design and Participants**

The trial methods have been previously described in detail.^9,20^ In summary, a randomised, controlled, single masked clinical trial was conducted in Ethiopia between Feb 13, 2014, and April 30, 2015. Adults with TT defined as one or more eyelashes touching the eye or evidence of epilation, identified from a community-based screening in districts of West Gojam Zone, Amhara Region, Ethiopia were examined for eligibility. People with trichiasis due to other causes, recurrent trichiasis after previous surgery, hypertension, pregnancy, and those under 18 years were excluded. Those eligible and consented to participate following a written informed consent in Amharic were enrolled. This report adhered to standard CONSORT guidelines.

**Randomisation and Masking**

Participants were randomly assigned (1:1) to either PLTR or BLTR surgery. Randomisation was stratified by surgeon and sequences were computer-generated by an independent statistician with random block sizes of 4 or 6. Allocations were concealed in sequentially numbered, sealed, opaque envelopes. Examiners who were responsible for clinical observations at baseline and follow-ups were masked to allocation. The surgery was performed by six experienced nurse/health officer trichiasis surgeons. These were already trained, certified, and regularly performing PLTR surgery. They were trained rigorously on the BLTR procedure using the WHO trichiasis surgery training manual,^2^ and were then re-standardised on both surgical procedures after six-months of regular practice.

**Procedures**

Participant eyes were examined (EH) at baseline prior to randomisation using 2·5× binocular loupes and torch, and graded using the detailed World Health Organisation (WHO) Follicles Papillae Cicatricae (FPC) Grading System.^21^ The number, location and type of trichiasis lashes, corneal scarring, and tarsal conjunctival scarring and inflammation were graded and recorded. Presenting distance logMAR (Logarithm of the Minimum Angle of Resolution) visual acuity was measured using PeekAcuity software on a Smartphone in a dark room.^22^ Four standardized high-resolution digital photographs of trichiasis, cornea, and tarsal conjunctiva were taken. After the randomisation, during the surgeries, intraoperative and immediate postoperative observations were made to measure incision length, height and regularity by three trained nurses. Number of scissor cuts made to make an adequate dissection medially and laterally. The number, spacing and tension of the mattress sutures were recorded.

Participants were re-examined at 10-days, 6-months, and 12-months postoperatively, following the same assessment procedures as per baseline. The only additional elements were assessment for granuloma, level of eyelid correction, and post-operative eyelid contour abnormalities (ECA). ECA were graded according to the PRET trial methodology,^23^ and grouped for analysis: (1) clinically non-significant ECA, which included mild ECA; and (2) clinically significant ECA, which included moderate-to-severe ECA.

The four-year follow-up was approved by the Ethiopian National Health Research Ethics Review Committee, the London School of Hygiene & Tropical Medicine Ethics Committee, Emory University Institutional Review Board, and the Ethiopian Food, Medicine and Healthcare Administration and Controls Authority. The study was conducted in compliance with the Declaration of Helsinki and International Conference on Harmonisation–Good Clinical Practice.

Trial participants were re-contacted and invited to attend a four-year follow-up assessment at a local health facility. Those who were not able to come to the health facilities were examined in their homes. Reasons for loss to follow-up were identified and documented. Written informed consent in Amharic had been obtained at baseline, before the initial enrolment from participants. The participants were re-consented at the four-year follow-up. If a participant was unable to read and write, the information sheet and consent form were read to them and their consent recorded by thumbprint.

Participants were asked about any repeat surgery, epilation in the last 6-month, and satisfaction with their surgical outcome. They were examined following the same procedure as outlined above. Outcome assessment was conducted by an independent examiner (BA) who was masked to the intervention allocation and who had no prior involvement in randomisation allocation, outcome assessment, and data analysis. The four-year examiner received rigorous training and was standardised with the baseline and 12-month outcome assessor (EH). They had very strong agreement for the primary outcome (k=0.98).

**Outcomes**

Prior to the start of the 4-year follow-up, a single primary end point was prespecified in the approved protocol. The primary end point was postoperative TT at 4-years analysed as the proportion of individuals who developed one or more lashes touching the eye or clinical evidence of epilation at the 4-year examination, or a history of repeat surgery in the 4-year period. The secondary analysis of the primary outcome measure was cumulative PTT defined as the proportion of individuals who had developed PTT by 4-years, defined as one or more lashes touching the eye or clinical evidence of epilation at all follow-ups (10 day, 6- and 12-months, and 4-year), or a history of repeat surgery in the 4-year period.

Secondary outcome measures included: PTT difference by surgeon and baseline disease severity; under correction; eyelid contour abnormality prevalence and regression at 4-year, corneal opacity and vision changes; effect of PTT on corneal opacity and vision changes, factors influencing long term outcomes; and patient-reported outcomes.

**Statistical analysis**

Sample size determination was described in the 12-month results paper.^9^ Data were double-entered into Access 13 (Microsoft) and transferred to Stata 14 (StataCorp) for analysis. For participants who had bilateral surgery, the same randomly designated eye used for the 12-month analysis was used for the 4-year follow-up analysis (i.e. one eye only per participant included in analysis).

A modified intention-to-treat analysis was performed (modified meaning that participants who died during follow-up or were not seen at follow up for another reason were excluded). Otherwise all trial participants were analysed in the groups they were originally randomised and included in the analysis if they were seen at the 4-year follow-up (for the primary analysis) and at least at one follow-up time point (for the secondary analysis of the primary outcome). The effect of the intervention on primary outcome and binary secondary outcomes (cumulative PTT difference, PTT by baseline disease severity, under correction) was analysed using logistic regression to estimate the odds ratio (OR) and 95% CI. All comparisons between the two surgical procedures were controlled for surgeon as a fixed effect in the model, to account for the stratified randomisation. Vision and corneal opacity changes between baseline and 4-years were categorised as worse, same, better. Effect of the intervention on ordered categorical secondary outcomes (changes in visual acuity and corneal opacity, and patient-reported outcomes) were analysed using ordinal logistic regression. Intervention effects on ECA (categorical variable) prevalence and regression at 4-year were analysed using multinomial logistic regression to estimate relative risk ratio (RRR) and 95% CI. A non­-prespecified sign test was used to analyse if ECA regression between 6- and 12-month, and 12-month and 4-year is statistically significant in all study participants. In order to identify potential predictors of PTT at 4 years, first a univariable logistic regression was performed using PTT at 4 years as an outcome and factors with possible association with PTT (covariates) as exposures and was done separately for each intervention arm, before including all covariates that were associated with the outcome with p<0.2 into a multivariable model. Likelihood ratio test was used to decide on the covariates that should be included in the final multivariable model to determine the best fitting predictive model of risk factors for PTT at 4 years.

The trial was registered on the Pan African Clinical Trials Registry (PACTR201401000743135) and overseen by independent data and safety monitoring committee.

**Role of the funding source**

The funder of this long-term follow-up had no role in study design, data collection, data analysis, data interpretation, or writing of the report. The corresponding author had full access to all the data in the study and had final responsibility for the decision to submit for publication.

**Results**

Between Feb 13, 2014, and May 31, 2014, 5168 people were examined for eligibility, of whom 4166 were ineligible and 1002 were eligible among which two (<1%) declined surgery. Thus, 1000 trichiasis cases consented, were enrolled, and randomly assigned: 501 in the BLTR group and 499 in the PLTR group), Figure 1. The 4-year follow-up was conducted between February 1 and May 22, 2018. Among the 1000 individuals enrolled at baseline, 943 (94·3%) were re-examined: 471/499 (94·4%) from the PLTR arm, and 472/501 (94·2%) from the BLTR arm. Reasons for loss of follow-up are shown in Figure 1. About 4·2% (42/1000) of the trial participants (2·0% in the PLTR, and 2·2% in the BLTR) had died during the 4-year period.

Baseline demographic and clinical characteristics of participants seen at 4-year were balanced between the two groups, table 1. The mean age was 46 years and the majority were women (76·2%). Trichiasis severity and phenotypes, tarsal conjunctival inflammations, vision, and corneal opacity were comparable between the two groups. At baseline, major TT (>5 lashes touching the eye) was present in similar proportions of the two arms (46% in PLTR, 47% in BLTR), and 76% of the trichiasis lashes were corneal in both arms. Higher proportion of cases in both arms had metaplastic only lashes (44·4% in PLTR, and 41·3% in BLTR). At four year, 87% of the trial participants with PTT were minor cases (1-5 lashes).

At the 4-year follow-up, PTT was observed in 169/943 (17·9%) study eyes, among which 129 (76·3%) had minor trichiasis, and 23 (13·6%) had previously received repeat surgery in the study eye between baseline and 4-year follow-up. PTT was more frequent in the BLTR arm (105/472 [22·2%]) than the PLTR arm (64/471 [13·6%]), after adjusting for surgeon the OR was 1·82 (95%CI 1·29–2·56; p=0·0006). The risk difference for recurrent trichiasis between BLTR and PLTR procedures was 8·6% (95%CI 3·8–13·5).

By four years, cumulative PTT had developed in 238/996 (23·9%) study eyes. PTT was more frequent in the BLTR arm (148/499 [29·7%]) than the PLTR arm (90/497 [18·1%]), after adjusting for surgeon the OR was 1·91 (95%CI 1·42–2·57; p<0·0001), with PTT risk difference of 11·6% (95%CI 6·3–16·7). The cumulative PTT was greater in the BLTR arm than the PLTR arm at all follow-up time points (6-month, 12-month, and 4-year), Figure 2.

The secondary outcomes, all adjusted for surgeon effect, are shown in table 2. The risk of PTT between surgeons ranged between 11·1% and 17·7% in the PLTR group, and 19·2% and 28·1% in the BLTR group. There was more under-correction in the BLTR group than the PLTR group (15·6% vs 20·8%). Most of the under-correction tends to be peripheral in both the PLTR (65/73 [89·0%]) and BLTR (87/98 [88·8%]) surgeries. PLTR surgery had a lower risk of PTT in cases with baseline major TT than the BLTR surgery (17·8% vs 31·5%), and performs better across all severity of entropion. Participants were also asked about their satisfaction on the surgery. Comparable proportion of participants in both treatment arms reported satisfaction with the effect of the surgery on the trichiasis (93·0% PLTR and 92·6% BLTR), and the cosmetic appearance of the operated eyelid (96·2% for both surgeries). There was no evidence of a difference in logMAR visual acuity score changes (p=0·26), and corneal opacity grade changes (p=0·92) from baseline to 4-year between the two intervention arms.

The BLTR surgery had a lower risk of ECA than the PLTR surgery (3·6% vs 6·8%; RRR 0·52 [95% CI, 0·28–0·95]; p=0·033). But there was no statistically significant difference in the risk of clinically significant ECA between the two groups (4·0% vs 4·9%), table 2. The change in ECA regression is presented in table 3. There was also no statistically significant difference in regression of ECA between the PLTR and BLTR both between 6- and 12-months; and between 1- and 4-years. However, among the mild ECA diagnosed at 12-month follow-up, 81·6% in the PLTR group, and 76·1% in the BLTR group regressed to normal at 4-year (sign test p<0·0001). Similarly, among the clinically significant ECA diagnosed at 12-month, 48·3% in the PLTR group, and 51·5% in the BLTR group regressed to normal or mild ECA (sign test p<0·0001). At 4-year, 51·7% and 48·5% of cases with clinically significant ECA at 12-month remained unchanged in the PLTR and BLTR arm respectively.

There was strong evidence that major trichiasis, conjunctival scar severity, and any under correction at any location measured at immediate post-op during the baseline surgery independently predicted PTT 4-year after PLTR surgery. Increased number of peripheral dissections with scissors intraoperatively in the PLTR surgery at baseline had a long-term protective effect on postoperative TT. In the BLTR group, there was strong evidence that major trichiasis, mixed trichiasis lash location, and central under correction at immediate post-op at baseline independently predicted PTT 4-year after BLTR surgery, table 4.

Demographic and clinical factors (age, gender, trichiasis severity, entropion severity, conjunctival scarring, surgeon effect, number of scissor cuts during dissection, number of suture notes, suture knot symmetry, suture tension irregularity) that may predict ECA regression at 4-year were analysed by intervention group and for all participants (data not provided) and there was not strong evidence found of an association of any of these with ECA regression.

**Figure 1: Trial Profile**

**Table 1: Demographic and Clinical Characteristics of Cases Seen At 4-year Follow-Up**

|  | | **Treatment groups at baseline** | | | | **Participants seen at 4-years** | | | | | | | |
| --- | --- | --- | --- | --- | --- | --- | --- | --- | --- | --- | --- | --- | --- |
|  |  |  |  |  |  | **Baseline** | | | | **4-year** | | | |
| **Characteristic** | | **PLTR** | | **BLTR** | | **PLTR** | | **BLTR** | | **PLTR** | | **BLTR** | |
|  |  | **n/499** | **(%)** | **n/501** | **(%)** | **n/471** | **(%)** | **n/472** | **(%)** | **n/471** | **(%)** | **n/472** | **(%)** |
|  | |  |  |  |  |  |  |  |  |  |  |  |  |
| ***Gender (female)*** | | 388 | (77·8) | 377 | (75·2) | 366 | (77·7) | 353 | (74·8) | - | - | - | - |
|  | |  |  |  |  |  |  |  |  |  |  |  |  |
| ***Age (mean, SD)*** | | 47.2 | 15·0 | 47·5 | (14.9) | 46·6 | (14·4) | 46·8 | (14·4) | - | - | - | - |
| ***Trichiasis severity*** | |  |  |  |  |  | |  |  |  |  |  |  |
|  | No trichiasis | - |  | - |  | - | - | - | - | 407 | (86·4) | 367 | (77·8) |
|  | Minor (1–5) | 267 | (53·5) | 258 | (51·5) | 252 | (53·5) | 250 | (53·0) | 48 | (10·2) | 81 | (17·2) |
|  | Major (6–9) | 232 | (46·5) | 243 | (48·5) | 219 | (46·5) | 222 | (47·0) | 5 | (1·1) | 12 | (2·5) |
|  | Repeat surgery* | - | - | - | - | - | - | - | - | 11 | (2·3) | 12 | (2·5) |
|  | ***Median (IQR)†*** |  |  |  |  | 3 | (2–6) | 3 | (2–6) | 1 | (1–2) | 2 | (1–4) |
| ***Trichiasis lash location*** | |  |  |  |  |  |  |  |  |  |  |  |  |
|  | No lashes | - |  | - |  | - | - | - | - | 407 | (86·4) | 367 | (77·8) |
|  | Epilating | 38 | (7·6) | 44 | (8·8) | 38 | (8·1) | 40 | (8·5) | 5 | (1·1) | 14 | (3·0) |
|  | Corneal | 383 | (76·7) | 376 | (75·0) | 359 | (76·2) | 361 | (76·5) | 28 | (5·9) | 46 | (9·7) |
|  | Medial | 3 | (0·6) | 0 | (0·0) | 3 | (0·6) | 0 | (0·0) | 8 | (1·7) | 8 | (1·7) |
|  | Lateral | 8 | (1·6) | 5 | (1·0) | 8 | (1·7) | 4 | (0·8) | 4 | (0·8) | 5 | (1·1) |
|  | Corneal + Peripheral | 67 | (13.4) | 76 | (15.2) | 63 | (13·4) | 67 | (14·2) | 8 | (1·7) | 20 | (4·2) |
|  | Repeat surgery | - | - | - | - | - | - | - | - | 11 | (2·3) | 11 | (2·5) |
| ***Trichiasis lash type*** | |  |  |  |  |  |  |  |  |  |  |  |  |
|  | No lashes | - | - | - | - | - | - | - | - | 407 | (86·4) | 367 | (77·8) |
|  | Epilating | 38 | (7·6) | 44 | (8·8) | 38 | (8·1) | 40 | (8·5) | 5 | (1·1) | 14 | (3·0) |
|  | Entropic only | 126 | (25·2) | 117 | (23·3) | 121 | (25·7) | 111 | (23·5) | 19 | (4·0) | 32 | (6·8) |
|  | Metaplastic only | 224 | (44·9) | 206 | (41·1) | 209 | (44·4) | 195 | (41·3) | 24 | (5·1) | 35 | (7·4) |
|  | Misdirected only | 9 | (1·8) | 14 | (2·8) | 9 | (1·9) | 14 | (3·0) | 1 | (0·2) | 2 | (0·4) |
|  | Mixed | 102 | (20·4) | 120 | (23·9) | 94 | (20·0) | 112 | (23·7) | 4 | (0·8) | 10 | (2·1) |
|  | Repeat surgery |  |  |  |  | - | - | - | - | 11 | (2·3) | 12 | (2·5) |
| ***Tarsal conjunctiva inflammation ^a^*** | |  |  |  |  |  |  |  |  |  |  |  |  |
|  | None (P0) | 6 | (1·2) | 9 | (1·8) | 5 | (1·1) | 9 | (1·8) | 9 | (1·9) | 7 | (1·5) |
|  | Mild (P1) | 117 | (23·4) | 131 | (26·1) | 109 | (23·1) | 121 | (25·6) | 239 | (50·8) | 220 | (46·6) |
|  | Moderate (P2) | 306 | (61·3) | 297 | (59·3) | 294 | (62·4) | 281 | (59·5) | 208 | (44·3) | 228 | (48·3) |
|  | Severe (P3) | 70 | (14·0) | 64 | (12·8) | 63 | (13·4) | 61 | (12·9) | 14 | (3·0) | 17 | (3·6) |
| ***Best corrected logMAR VA in study eye*** | |  |  |  |  | 63 |  |  |  |  |  |  |  |
|  | -0·1 to 0·3 | 141 | (28·7) | 137 | (27·3) | 138 | (29·3) | 135 | (28·6) | 171 | (36·3) | 157 | (33·3) |
|  | 0·3 to 0·7 | 190 | (38·1) | 209 | (41·7) | 183 | (38·8) | 200 | (42·4) | 186 | (39·5) | 205 | (43·4) |
|  | 0·7 to 1·1 | 107 | (21·4) | 103 | (20·6) | 102 | (21·7) | 93 | (19·7) | 72 | (15·3) | 76 | (16·1) |
|  | 1·1 to 2·0 | 18 | (3·.6) | 18 | (3·6) | 15 | (3·2) | 18 | (3·8) | 13 | (2·8) | 5 | (1·1) |
|  | CF/HM/PL | 37 | (7·4) | 27 | (5·4) | 30 | (6·4) | 20 | (4·2) | 24 | (5·1) | 22 | (4·7) |
|  | NPL | 6 | (1·2) | 7 | (1·4) | 3 | (0·6) | 6 | (1·3) | 3 | (0·6) | 5 | (1·1) |
|  | Not taken | - | - | - | - |  | - | - | - | 2 | (0·4) | 2 | (0·4) |
| ***Corneal opacity ^b^*** | |  |  |  |  | - |  |  |  |  |  |  |  |
|  | None (CC0) | 121 | (24·2) | 132 | (26·3) | 114 | (24·2) | 128 | (27·1) | 137 | (29·1) | 129 | (27·6) |
|  | Peripheral (CC1) | 204 | (40·9) | 201 | (40·1) | 193 | (40·9) | 184 | (38·9) | 57 | (12·1) | 62 | (13·2) |
|  | Off centre faint (CC2a) | 94 | (18·8) | 94 | (18·7) | 90 | (19·1) | 91 | (19·3) | 92 | (19·6) | 97 | (20·7) |
|  | Off centre dense (CC2b) | 19 | (3·8) | 11 | (2·2) | 19 | (4·0) | 10 | (2·1) | 6 | (1·3) | 8 | (1·7) |
|  | Central faint (CC2c) | 48 | (9·6) | 50 | (10·0) | 44 | (9·3) | 47 | (10·0) | 150 | (31·9) | 147 | (31·4) |
|  | Central dense (CC2d) | 7 | (1·4) | 7 | (1·4) | 6 | (1·3) | 7 | (1·5) | 23 | (4·5) | 21 | (4·5) |
|  | Total central dense (CC3) | 4 | (0·8) | 6 | (1·2) | 4 | (0·8) | 5 | (1·1) | 3 | (0·6) | 4 | (0·8) |
|  | Phthisis (CC4) | 2 | (0·4) | 0 | (0·0) | 1 | (0·2) | 0 | (0·0) | 2 | (0·4) | 0 | (0·0) |

** 2 participants had repeat surgery between baseline and 12-month (one from each arm).*

*^a^ One missing value in the PLTR arm. ^b^ Five missing values (one in the PLTR, four in the BLTR).*

**Figure 2: Cumulative Postoperative Trachomatous Trichiasis (PTT) in 4-years by Intervention Arm**

Cummulative Proportion

|  | PLTR | BLTR |
| --- | --- | --- |
| TIME SINCE RANDOMISATION Baseline | 0% | 0% |
| TIME SINCE RANDOMISATION 10-day | 0.2% | 0.4% |
| TIME SINCE RANDOMISATION 6-month | 8.7% | 14.5% |
| TIME SINCE RANDOMISATION 12-month | 12.7% | 22.2% |
| TIME SINCE RANDOMISATION 4-year | 18.1% | 29.7% |

| ***Number at risk*** | | | | | |
| --- | --- | --- | --- | --- | --- |
| ***PLTR*** | ***499*** | ***499*** | ***498*** | ***456*** | ***436*** |
| ***BLTR*** | ***501*** | ***501*** | ***499*** | ***430*** | ***391*** |
| OR 1·91 (95% CI 1·42–2·57); p<0·0001 | | | | | |

**Table 2: Secondary Clinical and Patient Reported Outcomes**

| **Variables and clinical outcomes** | | **PLTR** | | **BLTR** | | **OR** | **(95% CI)** | **P value** |
| --- | --- | --- | --- | --- | --- | --- | --- | --- |
|  |  | **n/N** | **(%)** | **n/N** | **(%)** |  |  |  |
| ***Surgeon effect on PTT at 4 years ^a^*** | |  |  |  |  |  |  |  |
|  | 1 | 10/85 | (11·8) | 25/89 | (28·1) | 2·93 | (1·31–6·56) | 0·0089 |
|  | 2 | 10/90 | (11·1) | 18/90 | (20·0) | 2·00 | (0·87–4·61) | 0·10 |
|  | 3 | 14/79 | (17·7) | 15/78 | (19·2) | 1·10 | (0·49–2·48) | 0·81 |
|  | 4 | 12/85 | (14.1) | 20/91 | (22·0) | 1·71 | (0·78–3·76) | 0·18 |
|  | 5 | 6/46 | (13·0) | 10/45 | (22·2) | 1·90 | (0·63–5·77) | 0·25 |
|  | 6 | 12/86 | (13·9) | 17/79 | (21·5) | 1·69 | (0·75–3·81) | 0·20 |
| ***PTT by baseline trichiasis severity ^a^*** | |  |  |  |  |  |  |  |
|  | Minor TT | 25/252 | (9·9) | 35/250 | (14·0) | 1·50 | (0·87–2·61) | 0·15 |
|  | Major TT | 39/219 | (17·8) | 70/222 | (31·5) | 2·12 | (1·35–3·33) | 0·0011 |
| ***PTT by baseline entropion severity ^a^*** | |  |  |  |  |  |  |  |
|  | None/Mild | 11/99 | (11·1) | 22/85 | (25·9) | 2·87 | (1·26–6·55) | 0·012 |
|  | Moderate | 35/296 | (11·8) | 62/316 | (19·6) | 1·80 | (1·15–2·84) | 0·010 |
|  | Severe | 18/76 | (23·7) | 21/71 | (29·6) | 1·37 | (0·64–2·91) | 0·42 |
| ***Under correction in any part of the eyelid ^a^*** | |  |  |  |  |  |  |  |
|  | No | 396 | (84·4) | 373 | (79·2) | 1·44 | (1·03–2·02) | 0·034 |
|  | Yes | 73 | (15·6) | 98 | (20·8) |  |  |  |
| ***Eyelid contour abnormality at 4-yr ^b^*** | |  |  |  |  |  |  |  |
|  | None (base outcome) | 420 | (89·2) | 432 | (91·5) | 1 | - | - |
|  | Clinically None-significant (Mild) | 32 | (6·8) | 17 | (3·6) | 0·52 | (0·28–0·95) | 0·033 |
|  | Clinically Significant (Moderate and Severe) | 19 | (4·0) | 23 | (4·9) | 1·18 | (0·63–2·21) | 0·60 |
| ***Satisfaction with effect of surgery on the trichiasis at 4-yr ^c^*** | | |  |  |  |  |  |  |
|  | Satisfied | 438 | (93·0) | 436 | (92·6) | 1·06 | (0·65–1·74) | 0·81 |
|  | Neither satisfied nor dissatisfied | 8 | (1·7) | 9 | (1·9) |  |  |  |
|  | Dissatisfied | 25 | (5.3) | 26 | (5·5) |  |  |  |
| ***Satisfaction with the cosmetic appearance at 4-yr ^c^*** | | |  |  |  |  |  |  |
|  | Satisfied | 453 | (96·2) | 453 | (96·2) | 1·00 | (0·51–1·96) | 0·99 |
|  | Neither satisfied nor dissatisfied | 6 | (1·3) | 6 | (1·3) |  |  |  |
|  | Dissatisfied | 12 | (2·5) | 12 | (2·5) |  |  |  |
| ***Vision changes, baseline to 4-yr ^c^*** | |  |  |  |  |  |  |  |
|  | Worse | 146 | (31·1) | 127 | (27·0) | 1·15 | (0·90–1·45) | 0·26 |
|  | Same | 138 | (33·7) | 167 | (35·5) |  |  |  |
|  | Better | 165 | (35·2) | 176 | (37·4) |  |  |  |
| ***Corneal opacity changes, baseline to 4-yr ^c^*** | |  |  |  |  |  |  |  |
|  | Worse (More opacity) | 222 | (47·2) | 223 | (47·6) | 0·99 | (0·77–1·26) | 0·92 |
|  | Same (No change) | 184 | (39·1) | 180 | (38·5) |  |  |  |
|  | Better (Less opacity) | 64 | (13·6) | 65 | (13·9) |  |  |  |

*^a^ Logistic regression analysis*

*^b^ Multinomial logistic regression analysis with Relative Risk Ratios (RRR)*

*^c^ Ordinal logistic regression analysis*

**Table 3: Longitudinal Eyelid Contour Abnormality Changes**

| **ECA grading change** | | **6-month to 12-month** | | | | | | **12-month to 4-year** | | | | | | |
| --- | --- | --- | --- | --- | --- | --- | --- | --- | --- | --- | --- | --- | --- | --- |
|  |  | **PLTR** | | **BLTR** | | **RRR** ^*^ | **95% CI** | **PLTR** | | **BLTR** | | **RRR** | | **95% CI** |
|  |  | **n/N** | **(%)** | **n/N** | **(%)** |  |  | **n/N** | **(%)** | **n/N** | **(%)** |  |  |  |
| **Clinically Non-Significant (CNS) ECA** | |  |  |  |  |  |  |  |  |  |  |  | |  |
|  | Mild to none | 18/77 | (23·4%) | 10/52 | (19·2%) | 1·18 | (0·47–2·96) | 71/87 | (81·6%) | 35/46 | (76·1%) | 0·70 | (0·30–1·62) | |
|  | Remain mild | 49/77 | (63·6%) | 23/52 | (44·2%) | base | - | 15/87 | (17·2%) | 10/46 | (21·7%) | base | - | |
|  | Mild to CS | 10/77 | (13·0%) | 19/52 | (36·5%) | 4·05 | (1·62–10·08) | 1/87 | (1·2%) | 1/46 | (2·2%) | - | - | |
| **Clinically Significant (CS) ECA** | |  |  |  |  |  |  |  |  |  |  |  | |  |
|  | CS to none | 0/20 | (0·0%) | 0/18 | (0·0%) | - | - | 7/29 | (24·1%) | 12/33 | (36·4%) | 1·71 | (0·54–5·48) | |
|  | CS to mild | 3/20 | (15·0%) | 2/18 | (11·1%) | 0·71 | (0·10–4·81) | 7/29 | (24·1%) | 5/33 | (15·1%) | 0·83 | (0·21–3·29) | |
|  | Remain CS | 17/20 | (85·0%) | 16/18 | (88·9%) | base | - | 15/29 | (51·7%) | 16/33 | (48·5%) | base | - | |
| **Overall Regression** | |  |  |  |  |  |  |  |  |  |  |  |  | |
|  | Remained the same or progressed | 76/97 | (78·4%) | 58/70 | (82·9%) | 1·77 | (0.86–3.64) | 31/116 | (26·7%) | 27/79 | (34·2%) | 1.42 | (0.76–2.64) | |
|  | Regressed/improved | 21/97 | (21·6%) | 12/70 | (17·1%) |  |  | 85/116 | (73·3%) | 52/79 | (65·8%) |  |  | |

*Note: Changes in eyelid contour abnormalities were assessed in participants seen at both at 12-month and 4-year follow-ups.*

** Relative Risk Ration from Multinomial logistic regression testing the effect of the intervention on ECA change between follow-up time points.*

**Table 4: Univariable and Multivariable Association of Factors with Postoperative TT At 4 Years, Stratified by Type of Surgery**

| **Demographic and Clinical Factors** | | | **PLTR (N=471)** | | | | | | | | **BLTR (N=472)** | | | | | | | |
| --- | --- | --- | --- | --- | --- | --- | --- | --- | --- | --- | --- | --- | --- | --- | --- | --- | --- | --- |
|  |  |  | **PTT** | |  | **Univariable Analysis** | | **Multivariable Analysis** | | | **PTT** | |  | **Univariable Analysis** | | **Multivariable Analysis** | | |
|  |  |  | **n/N** | **(%)** | ***OR*** | ***95% CI*** | ***p-value*** | ***OR*** | ***95% CI*** | ***p-value*** | **n/N** | **(%)** | ***OR*** | ***95% CI*** | ***p-value*** | ***OR*** | ***95% CI*** | ***p-value*** |
| **Age, yrs** | | |  |  |  |  |  |  |  |  |  |  |  |  |  |  |  |  |
|  | 18–29 | | 6/56 | (10·7%) | 1·11 | (0·93–1·34) | 0·24 | - | - | - | 7/46 | (15·2%) | 1·21 | (1·04–1·41) | 0·013 | 1·17 | (1·00–1·38) | 0·054 |
|  | 30–39 | | 7/82 | (8·5%) |  |  |  |  |  |  | 21/106 | (19·8%) |  |  |  |  |  |  |
|  | 40–49 | | 18/119 | (15·1%) |  |  |  |  |  |  | 23/105 | (21·9%) |  |  |  |  |  |  |
|  | 50–59 | | 16/105 | (15·2%) |  |  |  |  |  |  | 18/98 | (18·4%) |  |  |  |  |  |  |
|  | 60–69 | | 13/72 | (18·1%) |  |  |  |  |  |  | 20/78 | (25·6%) |  |  |  |  |  |  |
|  | 70 + | | 4/37 | (10·8%) |  |  |  |  |  |  | 16/39 | (41·0%) |  |  |  |  |  |  |
| **Trichiasis Severity** | | |  |  |  |  |  |  |  |  |  |  |  |  |  |  |  |  |
|  | | Minor | 25/252 | (9·9%) | 1·97 | (1·15–3·37) | 0·014 | 2·11 | (1·19–3·74) | 0·010 | 35/250 | (14·0%) | 2·83 | (1·79–4·46) | <0·0001 | 2·30 | (1·40–3·76) | 0·0009 |
|  | | Major | 39/219 | (17·8%) |  |  |  |  |  |  | 70/222 | (31·5%) |  |  |  |  |  |  |
| **Lash location** | | |  |  |  |  |  |  |  |  |  |  |  |  |  |  |  |  |
|  | | Epilating | 2/38 | (5·3%) | 0·38 | (0·09–1·58) | 0·18 | - | - | - | 9/40 | (22·5%) | 1·32 | (0·60–2·91) | 0·49 | 1·25 | (0·54–2·88) | 0·60 |
|  | | Corneal | 47/359 | (13·1%) | 1 | - | - |  |  |  | 65/361 | (18·0%) | 1 | - | - | - | - | - |
|  | | Peripheral | 1/11 | (9·1%) | 0·66 | (0·08–5·30) | 0·70 |  |  |  | 1/4 | (25·0%) | 1·52 | (0·15–14·8) | 0·72 | 1·88 | (0·18–19·6) | 0·60 |
|  | | Corneal +Peripheral | 14/63 | (22·2%) | 1·90 | (0·97–3·70) | 0·060 |  |  |  | 30/67 | (44·8%) | 3·69 | (2·12–6·41) | <0·0001 | 3·36 | (1·86–6·08) | 0·0001 |
| **Tarsal conjunctiva scar** | | |  |  |  |  |  |  |  |  |  |  |  |  |  |  |  |  |
|  | | Mild | 4/45 | (8·9%) | 1·81 | (1·06–3·11) | 0·030 | 1·86 | (1·05–3·31) | 0·034 | 8/51 | (15·7%) | 1·40 | (0·91–2·14) | 0·12 | - | - | - |
|  | | Moderate | 45/358 | (12·6%) |  |  |  |  |  |  | 77/348 | (22·1%) |  |  |  |  |  |  |
|  | | Severe | 15/68 | (22·1%) |  |  |  |  |  |  | 20/73 | (27·4%) |  |  |  |  |  |  |
| **Surgeon** | | |  |  |  |  |  |  |  |  |  |  |  |  |  |  |  |  |
|  | | 1 | 10/85 | (11·8%) | 1·07 | (0·42–2·71) | 0·40 | - | - | - | 25/89 | (28·1%) | 1·64 | (0·79–3·40) | 0·18 | - | - | - |
|  | | 2 | 10/90 | (11·1%) | 1 | - | - | - | - | - | 18/90 | (20·0%) | 1·05 | (0·49–2·25) | 0·90 | - | - | - |
|  | | 3 | 14/79 | (17·7%) | 1·72 | (0·72–4·13) | 0·22 | - | - | - | 15/78 | (19·2%) | - | - | - | - | - | - |
|  | | 4 | 12/85 | (14·1%) | 1·31 | (0·54–3·22) | 0·55 | - | - | - | 20/91 | (22·0%) | 1·18 | (0·56–2.50) | 0·66 | - | - | - |
|  | | 5 | 6/46 | (13·0%) | 1·20 | (0·41–3·54) | 0·74 | - | - | - | 10/45 | (22·2%) | 1·2 | (0·49–2·95) | 0·69 | - | - | - |
|  | | 6 | 12/86 | (13·9%) | 1·30 | (0·53–3·18) | 0·57 | - | - | - | 17/79 | (21·5%) | 1·15 | (0·53–2·51) | 0·72 | - | - | - |
| **No. of medial and lateral dissections, median (range)** | | |  |  |  |  |  |  |  |  |  |  |  |  |  |  |  |  |
|  | | No PTT | 1 | (0–26) | 0·77 | (0·63–0·95) | 0·014 | 0·68 | (0·53–0·88) | 0·0037 | 2 | (0–17) | 0·92 | (0·83–1·02) | 0·12 | 0·88 | (0·78–1·01) | 0·062 |
|  | | PTT | 0 | (0–4) |  |  |  |  |  |  | 2 | (0–9) |  |  |  |  |  |  |
| **Undercorrection at any part of the eyelid** | | |  |  |  |  |  |  |  |  |  |  |  |  |  |  |  |  |
|  | | No | 53/431 | (12·3%) | 2·91 | (1·36–6·20) | 0·0058 | 3·72 | (1·63–8·47) | 0·0018 | 96/441 | (21·8%) | 1·54 | (0·68–3·47) | 0·30 | - | - | - |
|  | | Yes | 11/38 | (28·9%) |  |  |  |  |  |  | 9/30 | (30·0%) |  |  |  |  |  |  |
| **Central undercorrection** | | |  |  |  |  |  |  |  |  |  |  |  |  |  |  |  |  |
|  | | Corrected | 54/407 | (13·3%) | 1 | - | - | - | - | - | 90/404 | (22·3%) | 1 | - | - | - | - | - |
|  | | Overcorrected | 5/54 | (9·3%) | 0·67 | (0·25–1·75) | 0·41 | - | - | - | 8/58 | (13·8%) | 0·56 | (0·26–1·22) | 0·14 | 0·50 | (0·22–1·16) | 0·10 |
|  | | Undercorrected | 5/10 | (50·0%) | 6·54 | (1·83–23·3) | 0·0038 | - | - | - | 7/10 | (70·0%) | 5·70 | (2·06–32·1) | 0·0028 | 8·73 | (2·02–37·7) | 0·0037 |
|  | |  |  |  |  |  |  |  |  |  |  |  |  |  |  |  |  |  |

***Note****: Analysis is done using logistic regression model. Factors with possible association with postoperative TT were tested in univariable analysis, and those with p<0*·*2 were included in the initial model. Then, likelihood ratio test was used to decide on variables to be included in the final multivariable model. Surgeon was included in the multivariable model regardless of significance level in both PLTR and BLTR but results not presented as there was no effect. Central undercorreciton was not included in the multivariable model for the PLTR to avoid collinearity with central undercorrection. In the PLTR, all listed in the table except age, and central undercorrection were included in the initial model. Then Lash location was excluded from the final model after likelihood ratio test. In the BLTR, all listed in the table except undercorrection at any part of the eyelid were included in the initial model. Then tarsal conjunctival scarring was excluded from the final model after likelihood ratio test. PTT= Postoperative Trachomatous Trichiasis.*

**Discussion**

It is possible that outcomes may change a few years after TT surgery. However, the data from this long-term follow-up indicate PLTR remains superior to the BLTR with a significantly lower risk of postoperative trichiasis both cross-sectionally at and cumulatively by 4-years after trichiasis surgery and is consistent with the one-year results we have previously reported.^9^ Moreover, PLTR still had a lower risk of PTT across all severity of trichiasis and entropion groups, and had lower risk of under-correction as was found at 1-year. These data support the WHO recommendation that new surgeons should be trained on the PLTR procedure for the programmatic management of TT.^15^ We have discussed in detail in the 1-year report why these outcome differences between the PLTR and the BLTR might have occurred.^9^ We believe the PLTR procedure provides a greater, more stable outward rotation of the distal portion of the eyelid.

The major factors predicting long-term outcomes were also similar to those reported at 1-year which included preoperative disease severity and surgical factors such as peripheral dissection and under-correction.^20^ Encouragingly, making adequate peripheral dissection still had a long-term protective effect on PTT in PLTR surgery indicating that it can be prevented with quality surgery and can be addressed easily during surgical trainings and supportive supervision. Special attention should also be provided for cases with advanced disease which should be operated by the most experienced surgeon available in the programme using the PLTR surgical procedure.

ECA has been a major issue for surgical programmes. It is cosmetically disfiguring, posing probably a greater concern than PTT for patients and surgeons. The good news is ECA, regressed between 6- and 12-months, and 1- and 4-years after surgery in about 73% and 66% of the cases in PLTR and BLTR surgeries respectively. However, about 50% of the clinically significant ECA cases at 12-month in both procedures remained un-changed. The regression seen in clinically significant ECAs between 6 and 12-month in the PLTR arm (15·0%) was much less than we have found in one of our recent trials which used the PLTR (40·6%).^10^ Rather the regression at 4-year in this trial (48·3%) was comparable to the regression at 12-month in the earlier trial. These results suggest that those with clinically significant disfiguring ECA need to be addressed surgically. However, in most trachoma endemic settings, neither highly skilled personnel which can correct ECA, nor a standard surgical procedure that can be used to correct ECA are available.

The strengths and limitations of this trial with regard to its design have been discussed in detail elsewhere.^9,20^ We managed to follow 94% of the trial participants 4 years after enrolment. Trichiasis surgeons operated in this trial received rigorous training and standardisation. Risk of unmasking posed a potential design limitation at the 6- and 12-month follow-ups in relation to possibility of visible skin scar from the BLTR surgery, which was addressed with independent photographic grading. However, this was not an issue in this long-term follow-up as no eyelid skin scar would be visible 4-years after surgery. Another potential limitation could have been unmasking of the outcome assessor as the randomisation code has been broken for earlier analyses. However, the outcome assessment was done by an independent assessor masked to allocation of intervention who had no involvement either in randomisation or data analysis.

Overall, there is strong evidence that PLTR remains superior to BLTR with reduced long-term risk of postoperative trichiasis supporting the current WHO guideline that the PLTR should be the procedure of choice for training new surgeons in the programmatic management of TT. Surgical programmes need to provide attention in improving outcomes and establish a system to comprehensively manage cases with poor surgical outcomes. The majority of PTT cases had five or less metaplastic lashes indicating that most of these cases can be treated with less invasive non-surgical methods. A relatively simple surgical procedure that can be used in trachoma endemic settings is needed to address un-resolving clinically significant ECA cases.

**Data Sharing Statement**

The Amhara Regional Health Bureau Ethics Committee requires that all data sharing requests are reviewed and approved by them before data can be shared. Data is available to any researcher under reasonable request. To facilitate the data access process please contact [ethics@lshtm.ac.uk](mailto:ethics@lshtm.ac.uk).

**Contributors**

Literature search: EH MJB

Study conception and design: EH ZT EKC DM MJB

Data collection: EH TW ZT BA BG

Statistical analysis: EH DM

Data interpretation: EH DM MJB

Drafting the manuscript: EH

Critical revision of the manuscript for important intellectual content: All authors

Obtained funding: EH MJB

Administrative, technical or material support: TW ZT BA BG AG KC

Study supervision: ZT BG MJB

**Declaration of interests**

We declare no competing interests.

**Acknowledgements**

This work received financial support from the Coalition for Operational Research on Neglected Tropical Diseases, which is funded at The Task Force for Global Health primarily by the Bill & Melinda Gates Foundation, by the United States Agency for International Development through its Neglected Tropical Diseases Program, and with UK aid from the British people. EH is supported by the Queen Elizabeth Diamond Jubilee Trust. MJB and the initial trial was funded by The Wellcome Trust (Grant Number 098481/Z/12/Z). We thank the trachoma control programme in Amhara National Regional State of Ethiopia, which is collaboration between the Regional Health Bureau and the Lions-Carter Center SightFirst Initiative; the research study team; the study participants; West Gojam Zone Health Office and the Woreda Health Offices.

**References**

1.	World Health Organization. Trachoma control: a guide for programme managers. Geneva, Switzerland: World Health Organization 2006.

2.	World Health Organization. Trichiasis Surgery for Trachoma. Geneva, Switzerland: World Health Organization, 2013.

3.	Rajak SN, Collin JR, Burton MJ. Trachomatous trichiasis and its management in endemic countries. *Survey of ophthalmology* 2012; **57**(2): 105-35.

4.	Reacher M, Huber M, Canagaratnam R, Alghassany A. A trial of surgery for trichiasis of the upper lid from trachoma. *British Journal of Ophthalmology* 1990; **74**(2): 109-13.

5.	Reacher M, Muñoz B, Alghassany A, Daar AS, Elbualy M, Taylor HR, Muñoz B. AA, Daar A. S., Elbualy M., Taylor H. R. A controlled trial of surgery for trachomatous trichiasis of the upper lid. *Archives of Ophthalmology* 1992; **110**(5): 667-74.

6.	Adamu Y, Alemayehu W. A randomized clinical trial of the success rates of bilamellar tarsal rotation and tarsotomy for upper eyelid trachomatous trichiasis. *Ethiopian medical journal* 2002; **40**(2): 107.

7.	Dhaliwal U, Monga P, Gupta V. Comparison of three surgical procedures of differing complexity in the correction of trachomatous upper lid entropion: a prospective study. *Orbit* 2004; **23**(4): 227-36.

8.	Nasr AM. Eyelid complications in trachoma. I. Cicatricial entropion. *Ophthalmic surgery* 1989; **20**(11): 800-7.

9.	Habtamu E, Wondie T, Aweke S, Tadesse Z, Zerihun M, Zewudie Z, Kello AB, Roberts CH, Emerson PM, Bailey RL, Mabey DCW, Rajak SN, Callahan K, Weiss HA, Burton MJ. Posterior lamellar versus bilamellar tarsal rotation surgery for trachomatous trichiasis in Ethiopia: a randomised controlled trial. *The Lancet Global Health* 2016.

10.	Habtamu E, Wondie T, Aweke S, Tadesse Z, Zerihun M, Gashaw B, Roberts CH, Kello AB, Mabey DCW, Rajak SN, Callahan EK, Macleod D, Weiss HA, Burton MJ. Oral doxycycline for the prevention of postoperative trachomatous trichiasis in Ethiopia: a randomised, double-blind, placebo-controlled trial. *The Lancet Global Health* 2018; **6**(5): e579-e92.

11.	Gower EW, West SK, Harding JC, Cassard SD, Munoz BE, Othman MS, Kello AB, Merbs SL. Trachomatous trichiasis clamp vs standard bilamellar tarsal rotation instrumentation for trichiasis surgery: results of a randomized clinical trial. *JAMA ophthalmology* 2013; **131**(3): 294-301.

12.	Rajak SN, Habtamu E, Weiss HA, Kello AB, Gebre T, Genet A, Bailey RL, Mabey DC, Khaw PT, Gilbert CE. Absorbable versus silk sutures for surgical treatment of trachomatous trichiasis in Ethiopia: a randomised controlled trial. *PLoS medicine* 2011; **8**(12): e1001137.

13.	Habtamu E, Burton M. Clearing the trichiasis backlog: experiences in Amhara, Ethiopia. *Community eye health* 2015; **28**(90): 38-.

14.	The Carter Center. Twentieth Annual Trachoma Control Program Review. March 18-20, 2019; Atlanta, USA.

15.	World Health Organization Alliance for the Global Elimination of Trachoma by 2020. Second Global Scientific Meeting on Trachomatous Trichiasis. Cape Town, South Africa, 4-6 November 2015.

16.	WHO Alliance for the Global Elimination of Trachoma by 2020. Eliminating Trachoma: Accelerating Towards 2020, 2016.

17.	Burton MJ, Bowman RJC, Faal H, Aryee EAN, Ikumapayi UN, Alexander NDE, Adegbola RA, West SK, Mabey DCW, Foster A, Johnson GJ, Bailey RL. Long term outcome of trichiasis surgery in the Gambia. *British Journal of Ophthalmology* 2005; **89**(5): 575-9.

18.	Burton MJ, Kinteh F, Jallow O, Sillah A, Bah M, Faye M, Aryee EAN, Ikumapayi UN, Alexander NDE, Adegbola RA, Faal H, Mabey DCW, Foster A, Johnson GJ, Bailey RL. A randomised controlled trial of azithromycin following surgery for trachomatous trichiasis in the Gambia. *British Journal of Ophthalmology* 2005; **89**(10): 1282-8.

19.	Khandekar R, Mohammed AJ, Courtright P. Recurrence of trichiasis: a long-term follow-up study in the Sultanate of Oman. *Ophthalmic Epidemiol* 2001; **8**(2-3): 155-61.

20.	Habtamu E, Wondie T, Aweke S, Tadesse Z, Zerihun M, Gashaw B, Wondimagegn GS, Mengistie HD, Rajak SN, Callahan K, Weiss HA, Burton MJ. Predictors of Trachomatous Trichiasis Surgery Outcome. *Ophthalmology* 2017; **124**(8): 1143-55.

21.	Dawson CR, Jones BR, Tarizzo ML. Guide to trachoma control in programmes for the prevention of blindness: Geneva, Switzerland; 1981.

22.	Bastawrous A, Rono HK, Livingstone IA, Weiss HA, Jordan S, Kuper H, Burton MJ. Development and Validation of a Smartphone-Based Visual Acuity Test (Peek Acuity) for Clinical Practice and Community-Based Fieldwork. *JAMA ophthalmology* 2015; **133**(8): 930-7.

23.	Gower EW, West SK, Cassard SD, Munoz BE, Harding JC, Merbs SL. Definitions and Standardization of a New Grading Scheme for Eyelid Contour Abnormalities after Trichiasis Surgery. *PLoS Negl Trop Dis* 2012; **6**(6): e1713.
